# Discovery of a “White-Gray-Opaque” Tristable Phenotypic Switching System in *Candida albicans*: Roles of Non-genetic Diversity in Host Adaptation

**DOI:** 10.1371/journal.pbio.1001830

**Published:** 2014-04-01

**Authors:** Li Tao, Han Du, Guobo Guan, Yu Dai, Clarissa J. Nobile, Weihong Liang, Chengjun Cao, Qiuyu Zhang, Jin Zhong, Guanghua Huang

**Affiliations:** 1 State Key Laboratory of Mycology, Institute of Microbiology, Chinese Academy of Sciences, Beijing, China; 2 Department of Molecular and Cell Biology, School of Natural Sciences, University of California, Merced, Merced, California, United States of America; 3 University of Chinese Academy of Sciences, Beijing, China; 4 State Key Laboratory of Microbial Resources, Institute of Microbiology, Chinese Academy of Sciences, Beijing, China; Duke University Medical Center, United States of America

## Abstract

Non-genetic phenotypic variations play a critical role in the adaption to environmental changes in microbial organisms. *Candida albicans*, a major human fungal pathogen, can switch between several morphological phenotypes. This ability is critical for its commensal lifestyle and for its ability to cause infections. Here, we report the discovery of a novel morphological form in *C. albicans*, referred to as the “gray” phenotype, which forms a tristable phenotypic switching system with the previously reported white and opaque phenotypes. White, gray, and opaque cell types differ in a number of aspects including cellular and colony appearances, mating competency, secreted aspartyl proteinase (Sap) activities, and virulence. Of the three cell types, gray cells exhibit the highest Sap activity and the highest ability to cause cutaneous infections. The three phenotypes form a tristable phenotypic switching system, which is independent of the regulation of the mating type locus (*MTL*). Gray cells mate over 1,000 times more efficiently than do white cells, but less efficiently than do opaque cells. We further demonstrate that the master regulator of white-opaque switching, Wor1, is essential for opaque cell formation, but is not required for white-gray transitions. The Efg1 regulator is required for maintenance of the white phenotype, but is not required for gray-opaque transitions. Interestingly, the *wor1/wor1 efg1/efg1* double mutant is locked in the gray phenotype, suggesting that Wor1 and Efg1 could function coordinately and play a central role in the regulation of gray cell formation. Global transcriptional analysis indicates that white, gray, and opaque cells exhibit distinct gene expression profiles, which partly explain their differences in causing infections, adaptation ability to diverse host niches, metabolic profiles, and stress responses. Therefore, the white-gray-opaque tristable phenotypic switching system in *C. albicans* may play a significant role in a wide range of biological aspects in this common commensal and pathogenic fungus.

## Abstract 2

This study describes a novel “white-gray-opaque” tristable phenotypic switching system in the human fungal pathogen *Candida albicans*, revealing additional complexity in this organism's ability to adapt to changing environments.

## Author Summary

The capacity of the yeast *Candida albicans* to grow in several cellular forms—a phenomenon known as phenotypic plasticity—is critical for its survival and for its ability to thrive and cause infection in the human host. In this study, we report a novel form of *C. albicans*, the “gray” phenotype, which may enhance fitness and confer an adaptive advantage for this important pathogenic yeast in certain host environments. The gray cell type, together with the previously discovered “white” and “opaque” cell types, forms a tristable phenotypic switching system. The three phenotypes differ in their cellular and colony appearance, their global transcriptional profiles, their production of secreted aspartyl proteinases (enzymes that degrade host tissues and release nutrients), and their virulence in different infection models. Moreover, gray cells exhibit a level of mating competency that is intermediate between that of white and opaque cells. We further demonstrate that two key transcriptional regulators, Wor1 and Efg1, play central roles in the regulation of the “white-gray-opaque” tristable transitions. Our study reveals a multi-stable and heritable switching system, indicating that the adoption of distinct morphological forms in response to environmental change could be much more elaborate than previously thought.

## Introduction

The ability of a single genotype to generate a number of different phenotypes in response to environmental stimuli, known as phenotypic plasticity, enables microorganisms to rapidly adapt to their changing environment and to survive and thrive in certain ecological niches. The human pathogenic yeast *C. albicans* can switch among several morphological phenotypes in response to a variety of environmental cues [1],[2]. The ability to grow in different morphological forms is critical for both its commensal lifestyle and its existence as a pathogen [3],[4]. The “white-opaque” transition is a well-known bistable phenotypic switching system in *C. albicans* [5]. White and opaque cells are two morphologically distinct cell types [6]. White cells are small and round and form white and shiny colonies on solid media, while opaque cells are larger and elongated and form flatter and rougher colonies. White and opaque cells also differ in their gene expression profiles, mating competency, and virulence characteristics [7],[8]. For instance, opaque cells can mate more efficiently and are better at cutaneous infections than white cells, while white cells are more virulent in systemic candidiasis [8].

The white and opaque phenotypes are heritable and stable for many generations of cell divisions [5],[6]. There does not appear to be a stable intermediate phase between white and opaque in *C. albicans* [5],[9], although transient intermediate phenotypes have been observed at the cellular level during the process of high temperature-induced transitions [10]. *C. tropicalis* and *C. dubliniensis*, two human fungal pathogens closely related to *C. albicans*, can also undergo white-opaque switching [11]–[13]. We recently observed an intermediate phase between the white and opaque phenotypes in *C. tropicalis* and proposed that the phenotypic switching system in this species may be tristable [13].

The white-opaque transition is regulated by the bistable expression of the master regulator gene *WOR1* [14]–[16], and therefore, there is not an intermediate phase between white and opaque phenotypes. In the laboratory strain SC5314 and its derivatives, the mating type locus (*MTL*) controls white-opaque switching via repressing *WOR1* expression by the **a**1-α2 complex [7],[14]. We have recently reported that a subset of clinical isolates of *C. albicans MTL* **a**/α heterozygous strains can undergo white-opaque switching when cultured in N-acetylglucosamine (GlcNAc)-containing media [17], which is thought to mimic the host environment. The key regulators, including Wor1, Wor2, Efg1, and Czf1, constitute an interlocking transcriptional circuit controlling white-opaque switching via positive and negative feedback loops [18].

In this study, we report a novel morphological phenotype of *C. albicans*, referred to as the “gray” phenotype. This phenotype is heritable but distinct from the previously identified white and opaque phenotypes in cellular and colony appearance, global gene expression profiles, secreted aspartyl proteinase (Sap) activities, and virulence characteristics. The gray phenotype, together with the white and opaque phenotypes, forms a novel tristable and heritable switching system in *C. albicans*. We further demonstrate that neither Wor1 nor Efg1 are required for the maintenance of the gray phenotype. Deletion of *WOR1* blocks white-to-opaque and gray-to-opaque transitions, but not white-gray transitions. Deletion of *EFG1* blocks opaque-to-white and gray-to-white transitions, but not gray-opaque transitions. Deletion of both *WOR1* and *EFG1* locks cells in the gray phenotype. Therefore, Wor1 and Efg1 may coordinately regulate the “white-gray-opaque” tristable phenotypic switching system in *C. albicans*.

## Results

### Discovery of the Gray Phenotype and the White-Gray-Opaque Tristable Switching System in *C. albicans*

**Figure 1:**
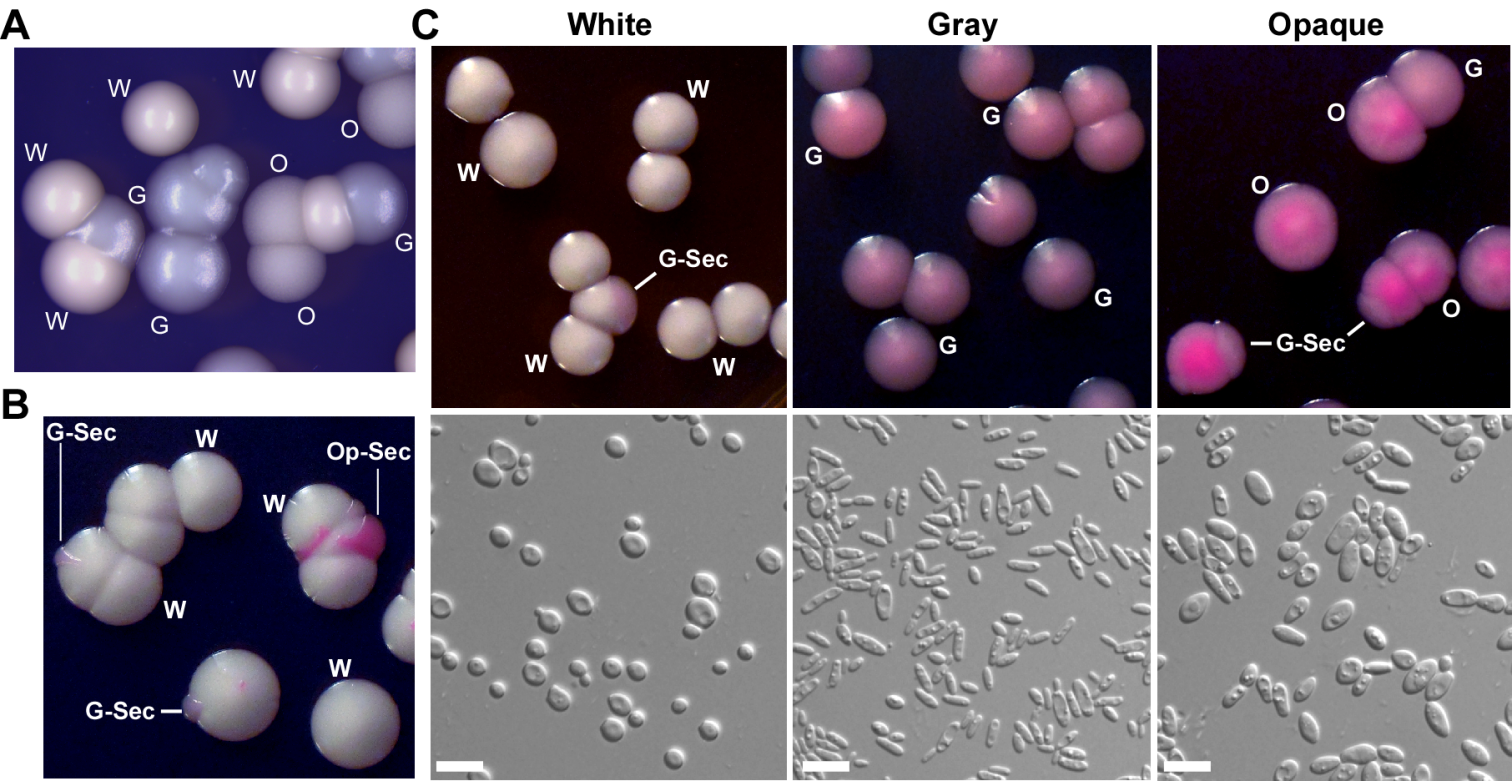
Three distinct phenotypes (white, gray, and opaque) of *C. albicans* on YPD medium. G, gray; G-Sec, gray sector; O, opaque; Op-Sec, opaque sector; W, white. The strain (BJ1097) was used. (A) Morphologies of white, gray, and opaque colonies on YPD agar without phloxine B. (B) Morphologies of white and sectored colonies on YPD agar containing phloxine B. A white colony initially grown on Lee's medium was replated onto YPD agar. The colonies were imaged after 5 days of growth at 25°C. The dye phloxine B stained opaque sectors dark pink and gray sectors light pink. (C) Colony and cellular morphologies of the three phenotypes of *C. albicans* on YPD agar with phloxine B. Colonies were grown at 25°C for 5 days. Scale bar, 10 µm.

We isolated a *C. albicans* strain (BJ1097) from the genital tract of a female patient at a women's health hospital in Beijing, China. We sequenced the internal transcribed spacers (ITS) and 5.8S rDNA region and verified that BJ1097 is a *C. albicans* strain. When this strain was grown on yeast extract-peptone-dextrose (YPD) agar plates for several days, we observed a novel colony phenotype, hereafter referred to as the “gray” phenotype, in addition to the typical white and opaque colony phenotypes (Figure 1A). Gray colonies appeared smooth and gray, while typical opaque colonies were gray and rough or “opaque,” and typical white colonies were relatively white and smooth. On YPD agar containing the dye phloxine B, the white colonies remained white and the opaque colonies were stained pink, while the gray colonies exhibited a distinctly lighter pink color (Figure 1B and 1C). The color of the gray colonies was noticeably different than that of the opaque colonies on phloxine B containing media. The cellular morphologies of the white, gray, and opaque phenotypes were also distinguishable on YPD medium (Figure 1C). Consistent with previous reports, white cells were round and small, while opaque cells were elongated and large. Gray cells were also elongated, but appeared much smaller than opaque cells (Figure 1C). The cellular and colony morphologies of the three phenotypes on Lee's glucose and Lee's GlcNAc medium are shown in Figures S1 and S2. Similar to the phenotypes on YPD medium, the order of coloration from darkest to lightest on Lee's media was opaque>gray>white. Cellular morphologies of white, gray, and opaque cells on Lee's media were also similar to those on YPD medium. The cellular morphology of gray cells was very similar to that of opaque cells of the haploid *C. albicans* strains recently reported by Hickman and colleagues [19]. We, therefore, performed fluorescence activated cell sorting (FACS) to assess ploidy, and found that all three cell types of BJ1097 are in fact diploid (Figure S1C).

**Figure 2:**
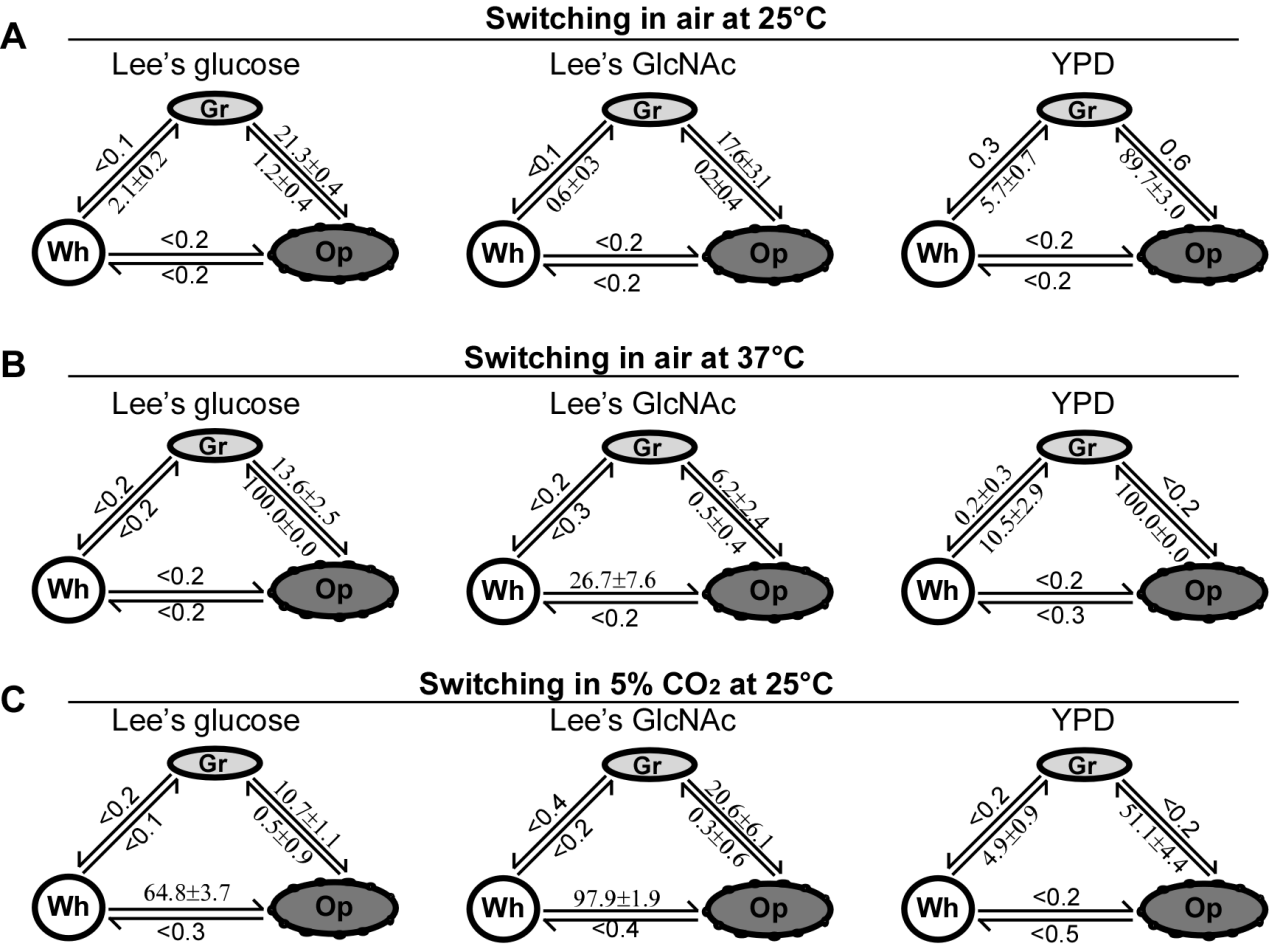
Switching frequencies of the white-gray-opaque tristable switching system in *C. albicans*. Gr, gray; Op, opaque; Wh, white. Colonies (strain BJ1097) were grown under the conditions indicated in the figure. Colonies were counted for switching frequency calculations. (A) Switching frequencies in air at 25°C for 5 days. (B) Switching frequencies in air at 37°C for 4 days. (C) Switching frequencies in 5% CO_2_ at 25°C for 5 days.

The switching frequencies of white-gray-opaque transitions in air at 25°C are shown in Figure 2A. On YPD medium plates, white-to-gray and opaque-to-gray switching frequencies were 5.7%±0.7% and 89.7%±3.0%, respectively, indicating that the white and especially opaque phenotypes are not stable under this culture condition. On Lee's glucose and Lee's GlcNAc media, the white and the opaque phenotypes were relatively stable when cultured in air at 25°C, while the gray-to-opaque switching frequencies were 21.3%±0.4% and 17.6%±3.1%, respectively. The white, gray, and opaque phenotypes were also stable in liquid Lee's media (Figure S3). Scanning electron microscopy (SEM) examinations demonstrated that the cell surfaces of white and gray cells were smooth, while at least a part of the opaque cell surface exhibited a pimpled appearance (Figure S4).

### White-Gray-Opaque Tristable Transitions at the Host Physiological Temperature (37°C)

As shown in Figure S5, the strain BJ1097 could switch among the white, gray, and opaque phenotypes in Lee's and YPD media at 37°C. Switching frequencies are shown in Figure 2B. On glucose containing media (YPD and Lee's glucose), opaque cells underwent a mass conversion to the gray phenotype (colony switching frequency = 100%). On Lee's GlcNAc medium, opaque cells were much more stable than on the other two media.

### Role of GlcNAc and CO_2_ in White-Gray-Opaque Tristable Transitions

GlcNAc and CO_2_ are white-to-opaque switching inducers, which are abundant in the host gut, a major niche of *C. albicans* [20],[21]. Consistent with our previous studies [17],[20],[21], the combination of GlcNAc and CO_2_ promoted white-to-opaque switching on Lee's medium (Figure 2C). On YPD medium, the induction effect of CO_2_ on the opaque phenotype was not obvious (Figures 2C and S6). The switching frequencies from opaque-to-gray were 89.7%±3.0% in air and 51.1%±4.4% in 5% CO_2_, suggesting that CO_2_ has an effect on stabilizing the opaque phenotype on YPD medium. In 5% CO_2_, gray cells are more stable than white cells, which showed a high frequency to switch to the opaque phenotype on Lee's glucose and Lee's GlcNAc media (Figure 2A and 2C).

At 37°C, opaque-to-gray switching on Lee's glucose and Lee's GlcNAc medium were 100% and 0.5%±0.4%, respectively, suggesting that GlcNAc can also stabilize the opaque phenotype (Figure 2B). However, neither GlcNAc nor CO_2_ had a notable effect on white-gray transitions on three different media both at 25°C and at 37°C (Figure 2A–2C).

### The Presence of the Gray Phenotype in Other Clinical Isolates of *C. albicans*

To test whether other clinical strains of *C. albicans* could form the gray phenotype, we plated 30 clinical isolates of *C. albicans* on Lee's media and cultured them at 25°C. These strains are all competent at white-opaque switching ([17] and our unpublished data). We found that a subset of strains could switch to the gray phenotype. Six examples are shown in Figure S7. The cellular morphology of gray cells of all these strains was similar to that of BJ1097. These results suggest that the white-gray-opaque transition is a general feature of clinical strains of *C. albicans*.

### Global Gene Expression Profiles in White, Gray, and Opaque Cells

**Figure 3:**
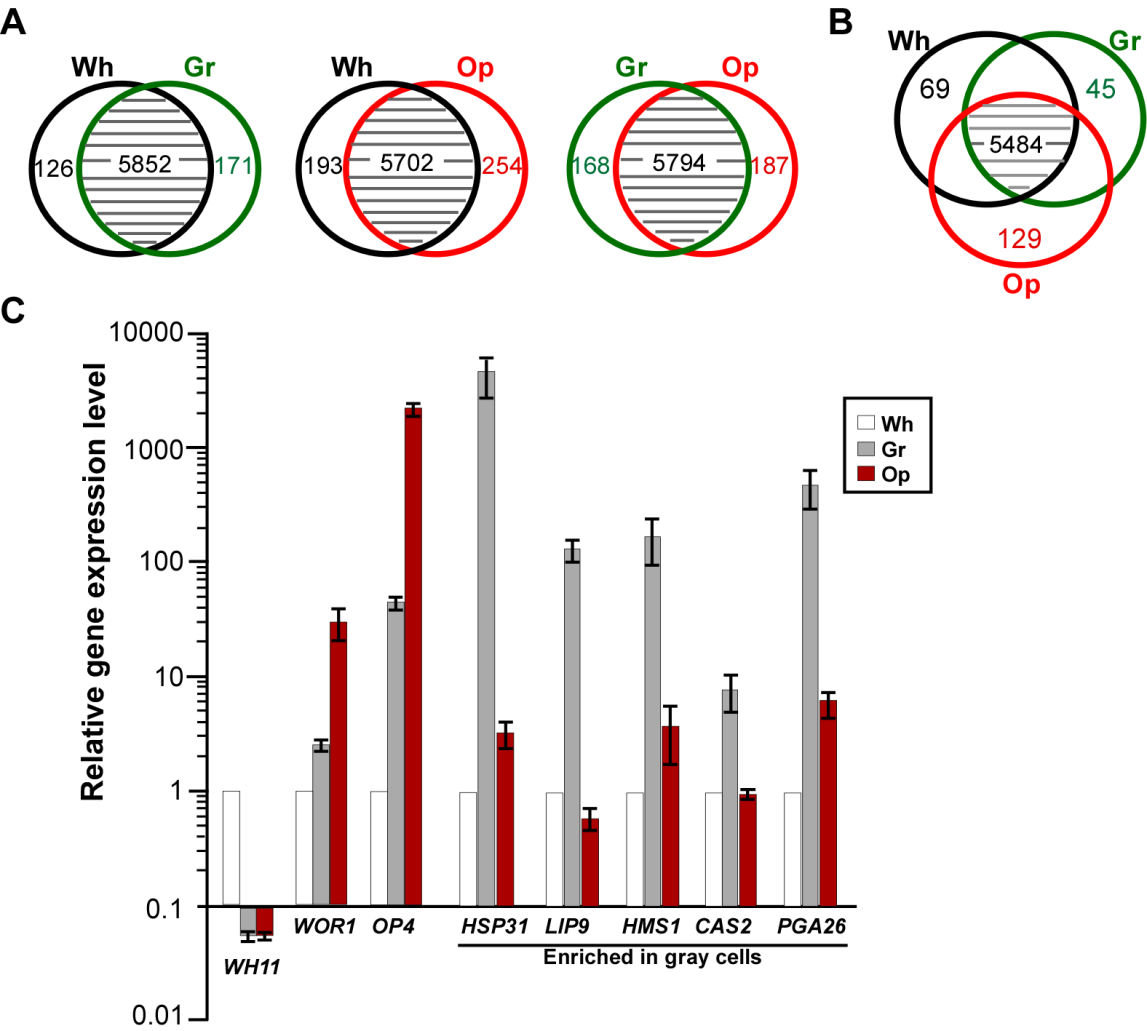
Differential gene expression profiles in white, gray, and opaque cells. Differentially expressed genes were defined as ones with greater than or equal to 4-fold difference of relative expression levels revealed by RNA-Seq analysis between two different cell types. The numbers of overlapping genes less than 4-fold difference of relative expression levels are also show. (A) Distinct gene expression patterns of white-gray, white-opaque, and gray-opaque cell types. The numbers indicate differentially expressed genes between the two cell types compared. (B) Distinct gene expression patterns of white, gray, and opaque cell types. The numbers indicate white (69)-, gray (45)-, or opaque (129)-enriched genes. (C) Verification of cell type-enriched genes by quantitative real-time PCR assays. *WH11*, white-enriched; *OP4* and *WOR1*, opaque-enriched; *HSP31*, *LIP9*, *HMS1*, *CAS2*, and *PGA26*, gray-enriched.

To better understand the differences among the three cell types, we performed RNA-Seq analysis to investigate their global gene expression profiles. As shown in Figure 3A and 3B, the three cell types exhibit distinct and overlapping gene expression patterns. A more detailed analysis of the differentially expressed genes in white, gray, and opaque cells are shown in Tables S1 and S2. Key findings are summarized as follows: (1) Gene expression profiles of white and opaque cells are consistent with our previous study performed in a different *MTL* **a**/α strain (CY110) and other reports in *MTL* homozygous strains [17],[22],[23]. For example, *WOR1*, *WOR3*, and *OP4* were enriched only in opaque cells, while *EFG1* was expressed in white and gray cells but not in opaque cells. *WH11* was enriched only in white cells. (2) Gray-enriched genes included cell wall-related (e.g., *PGA26* and *SUN41*), drug resistance-related genes (e.g., *CSA2*, *orf19.3348* and *orf19.3475*), stress-response-related (e.g., *HSPs*), metal ion metabolism-related genes (e.g., *FRE7* and *FRE30*), and some secreted enzymes (*SAPs* and *LIP9*). Notably, two oral infection-upregulated genes (*orf19.6200* [24] and *orf19.6070* [25]) were exclusively enriched in gray cells. (3) The expression profiles of metabolism-related genes, especially those involved in carbohydrate metabolism, exhibited distinct features from that of white and opaque cells. A small proportion of genes (<10%), such as the NADH oxidase gene *AOX2* and the glycerol permease gene *HGT10*, were highly expressed in gray cells. About 30% of metabolism-related genes showed a similar expression level in gray cells to that of white cells, ∼20% to that of opaque cells, and ∼30% exhibited an intermediate level between white and opaque. Consistent with the previous study [22], white cells express a fermentative metabolism-gene profile, while opaque cells adopt an oxidative one. These results indicate that gray cells may have a unique metabolic mode, which is different from that of white and opaque cells. (4) A large set of genes encoding signaling peptide- or glycosylphosphatidylinositol (GPI)-containing proteins were enriched in gray cells, including *orf19.3378*, *orf19.3376*, *orf19.3117*, *orf19.6200*, *PGA26*, and *IFF8*. (5) The absolute expression levels in opaque cells (indicated by the reads per kilobase per million mapped reads, or RPKM values) of *SAP* genes were much higher than those in white and gray cells. For example, the RPKM value of *SAP1* is over 20,000 in opaque cells, while it is less than 30 in both white and gray cells. Although the expression level of *SAP7* in gray cells is about 10-fold higher than that in opaque cells, the absolute expression levels in both cell types are very low (the RPKM values are 22 and 2 in gray and opaque cells, respectively).

### White, Gray, and Opaque Cells Exhibit Differential Secreted Aspartyl Protease (Sap) Activity

**Figure 4:**
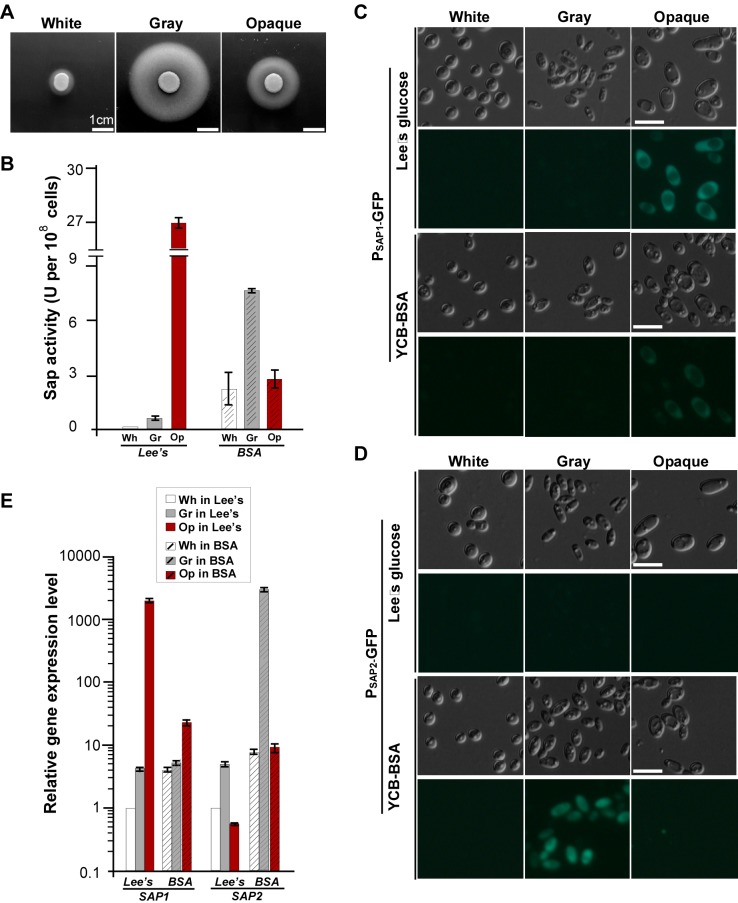
Differential Sap activities in white, gray and opaque cells. (A) Sap activities in white, gray, and opaque cells cultured on solid YCB-BSA medium. 5×10^6^ cells of each cell type in 5 µl ddH_2_O were spotted onto YCB-BSA medium plates and grown at 25°C for six days. The white precipitation zones (halos) around the cell spots indicate Sap-mediated BSA hydrolysis. Scale bar, 1 cm. (B) Sap activities in white, gray, and opaque cells cultured in liquid media. Cells were grown in liquid Lee's glucose or YCB-BSA medium. Quantitative activity assays are described in the Materials and Methods section. (C and D) Expression of GFP in the reporter strains of *SAP1p-GFP* (C) and *SAP2p-GFP* (D). Cells were grown on Lee's glucose and YCB-BSA plates for four days at 25°C in air. Scale bar, 10 µm. (E) Relative expression levels of *SAP1* and *SAP2* in white, gray, and opaque cells. Cells were grown in liquid Lee's glucose and YCB-BSA media for 24 hours at 25°C in air.

The transcriptional profiles of the *SAP* genes, which are known major virulence factors [26], were different in the three cell types. We therefore tested Sap activities using the yeast carbon base (YCB)-bovine serum albumin (BSA) medium assay. As expected, white cells showed lowest Sap activity, which is consistent with low expression levels of the *SAP* genes. However, we surprisingly found that gray cells exhibited higher Sap activity than opaque cells, indicated by the white halos of precipitated BSA (Figure 4A). Quantitative Sap activity assays verified these results (Figure 4B). This result is inconsistent with the expression profiles of *SAP* genes in gray and opaque cells. Since the RNA-Seq analysis was performed in Lee's glucose medium (without BSA), we predicted that the YCB-BSA medium induced the expression of *SAP* genes in gray cells and thus increased Sap activity. To test this hypothesis, we performed quantitative Sap activity assays. As predicted, the Sap activity of opaque cells was higher than that of gray cells in Lee's glucose medium, but was lower than that of gray cells in the YCB-BSA medium. Using a green fluorescent protein (GFP) reporter system (Figure 4C and 4D) and quantitative real-time PCR (Figure 4E) assays, we further found that *SAP1* was constitutively expressed in opaque cells in both media. *SAP2* exhibited extremely low expression levels in all three cell types in Lee's medium, while its expression level was increased over thousands of times in gray cells cultured in the YCB-BSA medium. Taken together, these results imply that the three cell types are likely to have differential virulence characteristics at different host niches.

### White, Gray, and Opaque Cells Differ in Virulence in a Murine Systemic Candidiasis Model and in Fungal Burdens at Different Host Organs

**Figure 5:**
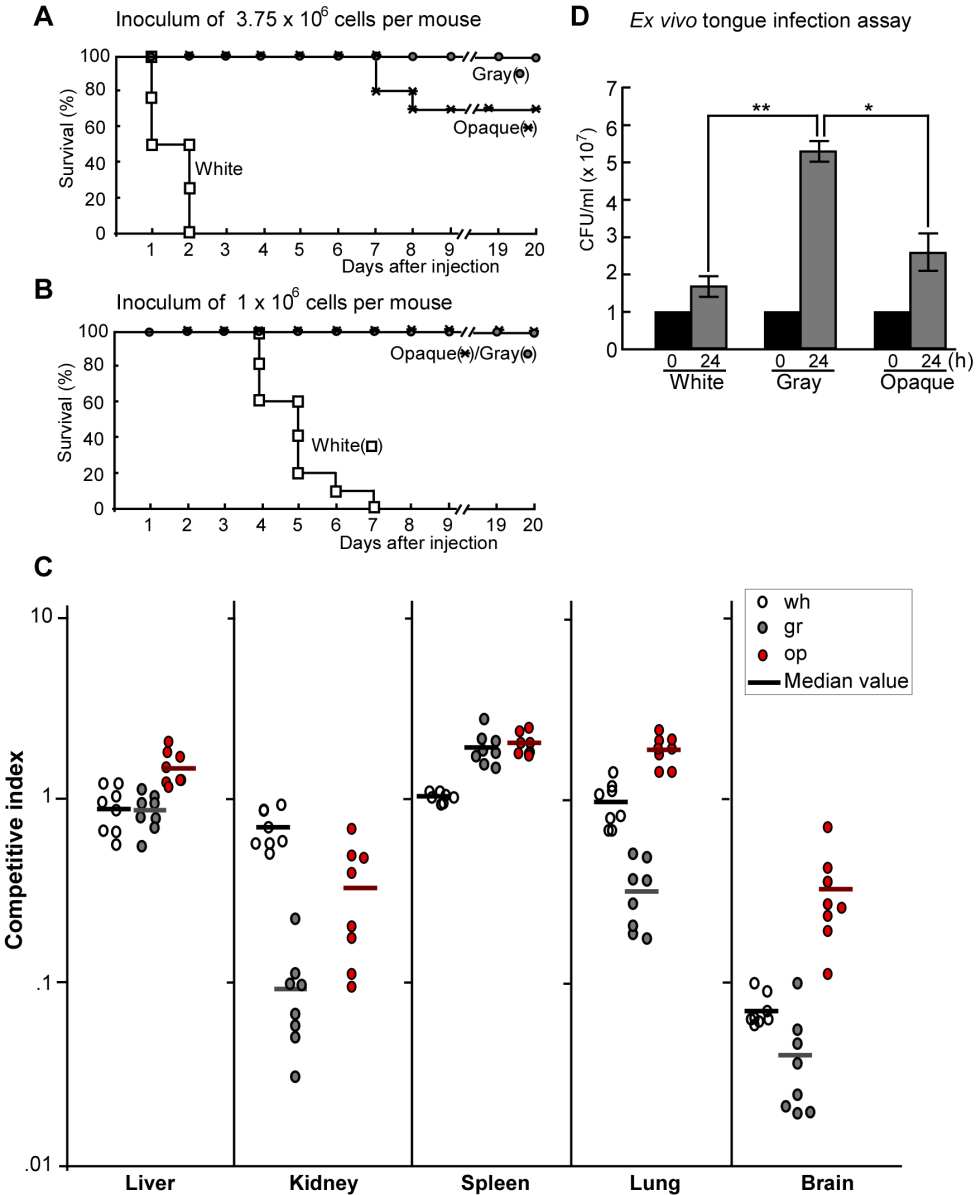
Virulence of white, gray, and opaque cells in systemic and cutaneous infections. (A and B) Survival curves for white, gray, and opaque cells of strain BJ1097. 3.75×10^6^ (A) or 1×10^6^ (B) cells of each cell type were injected into each mouse via tail vein. For each cell type, ten mice were used for infection. Survival rates with significant difference (*p*<0.05, Student's *t*-test, two tails): wh>op, wh>gr, and op>gr in (A); wh>op and wh>gr in (B). (C) White, gray, and opaque cells differ in fungal burdens in different organs in a mouse systemic infection model. 5×10^5^ cells of each type (white, gray, or opaque) of BJ1097 were mixed with 5×10^5^ cells of SC5314N (Nou^R^, locked in white phase) in 250 µl PBS and then injected into a mouse via tail vein. Mice were killed at 24 hours after injection. Different organs were used for fungal burden assays. Eight mice were used for each cell type. Competitive index = the ratio of CFU number of BJ1097 to CFU number of SC5314N in each organ. Each cycle represents a value of competitive index in a mouse (e.g., a white cycle represents the value of the ratio of BJ1097/SC5314N). Bar, median value. Competitive indexes with significant difference (*p*<0.01, Student's *t*-test, two tails): In the liver, op>wh and op>gr; in the kidney, wh>op and wh>gr; in the spleen, op>wh and gr>wh; in the lung, op>wh; and in the brain, op>wh and op>gr. Wh, white cells, gr, gray cells, and op, opaque cells. (D) Ex vivo tongue infection model. Tongues were excised from humanely killed mice and one tongue was added to each well of a 24-well polystyrene plate containing 1×10^7^ cells of white, gray, or opaque cells in 1 ml PBS. After 24 hours of incubation at 37°C, cells in the liquid and on the tongue (after homogenization) were plated onto YPD agar for CFU assays. The total cell number of each well (including cells in the liquid and attached to the tongue) is shown. “0 h” indicates initial inoculated cell number (1×10^7^) in each well. *p<0.05; **p<0.01 (Student's *t*-test, two tails). The experiment was repeated three times. For each time, three tongues were used for each cell type. The result of a representative experiment is shown.

White cells were much more virulent than gray and opaque cells in the survival assay of systemic infections (Figure 5A and 5B). At the higher inoculation concentration (3.75×10^6^ cells per mouse), the order of virulence observed from highest to lowest was white cells>opaque cells>gray cells (Figure 5A). At the lower inoculation concentration (1×10^6^ cells per mouse), both gray and opaque cells exhibited similarly low virulence, while white cells killed all the infected mice in seven days (Figure 5B). We further performed competitive infections to evaluate the fungal burdens in different organs. As shown in Figure 5C, the three types of cells differed in fungal burdens in different organs, suggesting that the three cell types may have distinct abilities to invade and colonize different host tissues. For example, the fungal burden of white cells was higher than that of gray and opaque cells in the kidney, while the fungal burdens of gray and opaque cells were relatively higher than those of white cells in the liver and spleen. The fungal burdens of opaque cells were significantly higher than those of gray cells in four (liver, kidney, lung, and brain) of the five organs examined. These results were consistent with the data of survival assays (Figure 5A).

### White, Gray, and Opaque Cells Differ in Virulence in Cutaneous Infection Models

Secreted extracellular proteinases (such as Sap enzymes and lipases) play critical roles in degrading host tissues, which is thought to help fungal growth by releasing nutrients as well as facilitate fungal penetration during infections [26]. As shown in the ex vivo tongue infection assay (Figure 5D), the order of growth rates for the three cell types from fastest to slowest was gray cells>opaque cells>white cells, indicating that gray cells are better suited for nutrient acquisition from the animal tissue. Consistently, SEM examinations at 48 hours post-inoculation revealed that gray and opaque cells caused significantly greater damage of the skin than white cells. The skin infected with white cells remained largely intact and smooth, while the skin infected with gray or opaque cells exhibited obvious degradation and damage (Figure S8).

### Transcription Factors, Wor1 and Efg1, Are Master Regulators of the White-Gray-Opaque Tristable Switching System

**Figure 6:**
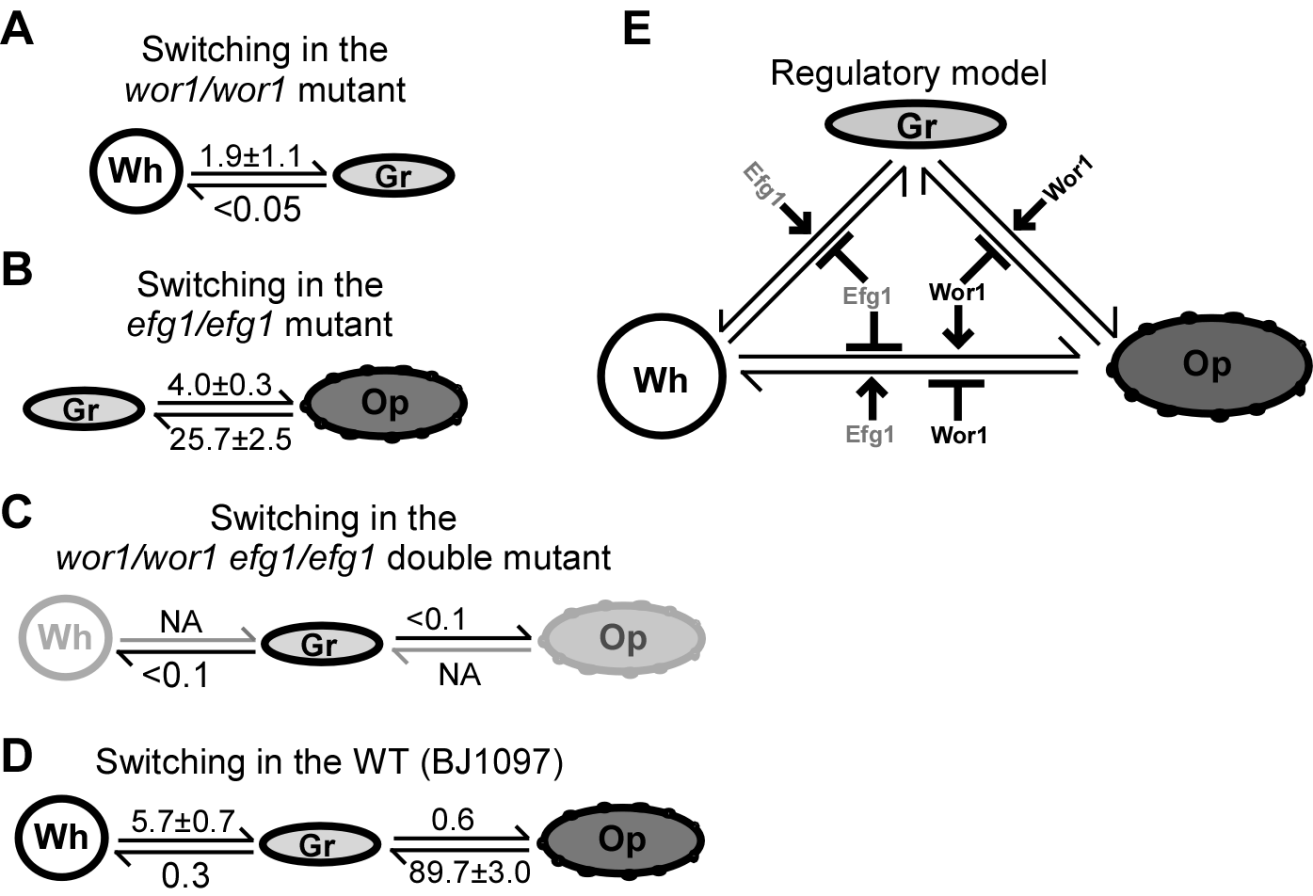
Roles of Wor1 and Efg1 in the regulation of white-gray-opaque transitions in *C. albicans*. NA, not available. The complete switching frequency data under different conditions are shown in Figure S10. (A) White-gray switching frequencies (%) on YPD medium. The *wor1/wor1* mutant cannot switch to the opaque phenotype under all conditions tested. (B) Gray-opaque switching frequencies (%) on YPD medium. The *efg1/efg1* mutant cannot switch to the white phenotype under all conditions tested. (C) Switching frequencies (%) on YPD medium. The *wor1/wor1 efg1/efg1* double mutant is locked in the gray phase under all conditions tested. (D) Switching frequencies (%) on YPD medium of the wild type control (adapted from Figure 2A). (E) Regulatory model of the white-gray-opaque tristable phenotypic switching system.

The basic morphology of gray cells is similar to that of opaque cells, although the former is much smaller in cell size (Figures 1, S1, and S2). Moreover, the key white-opaque switching regulator genes, *WOR1* and *EFG1*, were differentially expressed in the three cell types. We therefore set out to test whether the gray phenotype is governed by Wor1 and Efg1. Deletion of *WOR1* blocked opaque cell formation but allowed white-gray transitions (Figures 6A, S9, and S10). Deletion of *EFG1* blocked white cell formation but allowed gray-opaque transitions (Figures 6B, S9, and S10). The frequencies of white-gray switching in the *wor1/wor1* mutant and gray-opaque switching in the *efg1/efg1* mutant are shown in Figure S10. Given that Wor1 and Efg1 are essential for the formation of opaque and white cell types, respectively, we predicted that inactivating Wor1 and Efg1 simultaneously would block the formation of both the white and the opaque cell type and thus could only allow cells to exist in the gray phenotype. We, therefore, constructed a *wor1/wor1 efg1/efg1* double mutant. As predicted, the double mutant was locked in the gray phenotype and could not switch to the white or opaque phenotype under all culture conditions tested (Figures 6C, S10E, and S10F). The wild type (WT) control is shown in Figure 6D. These results indicate that (1) neither Wor1 nor Efg1 is essential for gray cell formation; (2) both Wor1 and Efg1 could repress the formation of the gray phenotype; (3) Wor1 and Efg1 may work coordinately in the regulation of the white-gray-opaque phenotype. A regulatory model of the tristable switching system by Wor1 and Efg1 is shown in Figure 6E.

### White-Gray-Opaque Transitions Are Independent of the *MTL*

**Figure 7:**
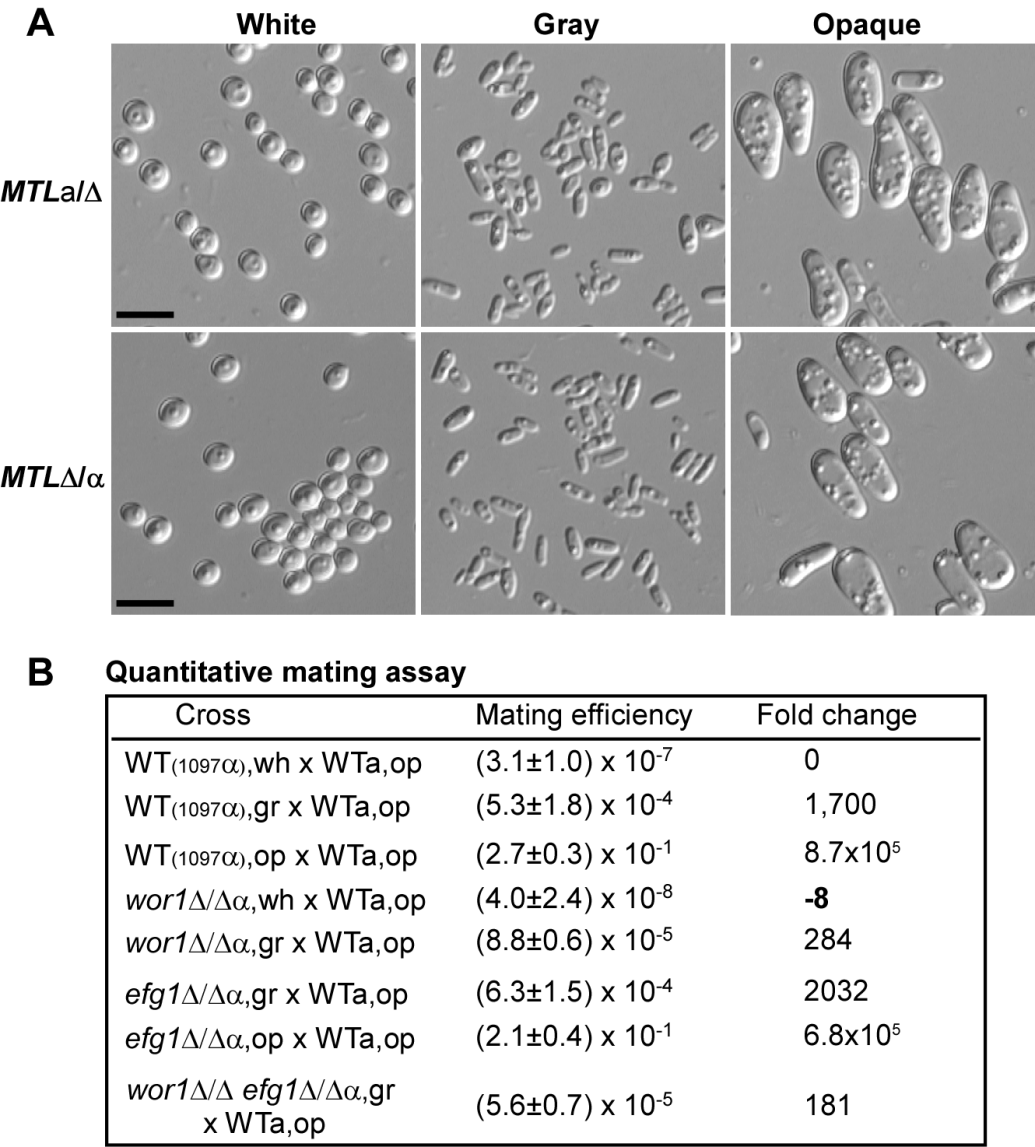
White-gray-opaque transitions are independent of the *MTL*. (A) Cellular morphologies of white, gray and opaque cells of BJ1097a (*MTL* **a**/Δ) and BJ1097α (*MTL*Δ/α). Scale bar, 10 µm. (B) White, gray, and opaque cells differ in mating efficiencies. Experimental strains with *MTL*Δ/α *URA3* ^+^ *sat1^−^* (or *Clon−*) genotype: the *wor1/wor1, efg1/efg1*, *wor1/wor1 efg1/efg1* double mutants, and the wild type BJ1097α. Tester strain WT**a** (*MTL* **a**/**a**, *ura3* ^−^ *Clon*+). Mating efficiency = average ± standard deviation (SD). The fold changes of mating efficiencies to that of WT white cells are also shown. The mean values were used for calculations.

Over 90% of clinical isolates of *C. albicans* are *MTL* heterozygotes (**a**/α). The strain BJ1097 and five strains used in Figure S7 are all *MTL* heterozygotes (**a**/α). We therefore examined whether the *MTL* locus regulates the gray phenotype. As shown in Figure 7A, both the *MTL* **a**/Δ and the Δ/α strains exhibited white, gray, and opaque phenotypes and could switch among the three phenotypes frequently (unpublished data). The cellular morphologies of gray cells of the *MTL* **a**/Δ or Δ/α were similar to those of the parent **a**/α gray cells. The cell size of opaque cells of the *MTL* **a**/Δ or Δ/α strain were larger than that of **a**/α opaque cells. More importantly, we found that the natural *MTL*α/α strain 19F could also undergo the white-gray-opaque transitions (Figure S7) [27]. Therefore, the white-gray-opaque tristable switching system is independent of the *MTL* locus.

### Gray Cells Exhibit an Intermediate Level of Mating Competency

We further demonstrated that gray cells mate over 1,000 times more efficiently than white cells, but hundreds of times less efficiently than opaque cells in the wild type strains (Figure 7B). The relatively high mating efficiency of gray cells could be partly due to their high switching frequency to the opaque phase (Figure 2A). To rule out this possibility and characterize the mating ability of gray cells, we next performed mating assays in the *wor1/wor1*, *efg1/efg1*, and *wor1/wor1 efg1/efg1* mutants. Gray cells of the *wor1/wor1* mutant mated about 2,000 times more efficiently than their white cell counterparts and 248 times more efficiently than white cells of the wild type. In the *efg1/efg1* mutant, gray cells exhibited a mating ability comparable to that of gray cells of the wild type. Consistently, the *wor1/wor1 efg1/efg1* double mutant, locked in the gray cell type, showed an intermediate mating competence between the white and the opaque phenotype (Figure 7B). These results suggest that gray cells indeed mate more efficiently than white cells since cells of the *wor1/wor1* and *wor1/wor1 efg1/efg1* mutants cannot switch to the highly mating-competent opaque phenotype.

## Discussion

High-frequency switching of colony morphology was observed in several clinical isolates of *C. albicans* by the Soll lab [28],[29]. The strains could switch heritably and reversibly between at least seven different phenotypes, not including the white-opaque transition [28],[29]. Here we report a novel morphological phenotype, the gray cell type, and a white-gray-opaque tristable switching system in *C. albicans*. Our new findings, together with previous reports [28],[29], suggest that *C. albicans* is capable of undergoing multiple-stable phenotypic transitions under certain environmental conditions. Compared with white and opaque cells, gray cells exhibit several unique characteristics: (1) distinct cellular and colony appearance; (2) high Sap activity in BSA-containing media; (3) tissue-specific infection ability; and (4) differential global gene expression profiles. A more comprehensive comparison of features of the three phenotypes is presented in Table 1.

**Table 1:** Comparison of features of white, gray, and opaque cell types.

Characteristics	Subcharacteristics	Cell Type		
		White	Gray	Opaque
**Colony appearance on solid media**	Without phloxine B	White and shiny	Gray and shiny	Gray and rough
	With phloxine B	White and shiny	Light pink and shiny	Dark pink and rough
**Cellular appearance**	—	Round and small	Elongated and small	Elongated and large
**Cell size** (in stationary phase)	Length/diameter (µm)	4.9–6.8	5.9–6.9	9.5–11.8
	Volume (µm^3^)	85–135	30–55	170–290
**Mating competency**	—	Low	Intermediate	High
**Virulence**	In systemic infections	High	Low	Low
	In cutaneous infections	Low	High	Intermediate
**Master regulator**	—	Efg1	NA	Wor1
**Cell type-specific genes**	—	*WH11, ALS2, ALS4, HGT19, etc.*	*LIP9, PGA26, HSP31, HMS1, etc.*	*OP4, SAP1, WOR1, PHO89, etc.*

These features were characterized based on examining the three cell types of BJ1097 grown in Lee's glucose medium.

NA, not available

### Environmental Cues Regulate White-Gray-Opaque Transitions

The tristable and heritable phenotypic switching system reported in this study may confer the pathogen with higher plasticity and capacity of environmental adaptation. White-gray-opaque transitions can both occur spontaneously and be induced by environmental cues. Although CO_2_ and GlcNAc promote white-to-opaque switching and stabilize the opaque phenotype [21], they have no obvious effect on the induction of the gray phenotype (Figure 2). However, the rich YPD medium facilitates the formation of gray cells (Figure 2). These results suggest that white and gray cells differ in response to environmental cues and that these three cell types may have different abilities to adapt to specific host niches.

### Phase-Specific Gene Expression Profiles, Saps, Virulence, and Niche Adaptation

White, gray, and opaque cells show distinct global gene expression profiles. The gray cell type-enriched genes include these encoding secreted enzymes, cell wall and surface proteins, metabolism- and antifungal-related proteins, and filamentation-regulators. This unique gene expression profile could contribute to a number of important biological traits of *C. albicans*, such as filamentation and biofilm formation, virulence, and resistance to antifungals. For example, a large set of genes encoding cell wall, membrane, and extracellular proteins, was upregulated in gray cells. These proteins directly interact with the extracellular environment and may play critical roles in sensing and responding to environmental changes.

Secreted proteinases, including Saps in *C. albicans*, play important roles in nutrient acquisition, tissue adherence, invasion, and infection [26]. Interestingly, although the expression of a number of *SAP* genes was only enriched in opaque cells in Lee's glucose medium, gray cells exhibited higher Sap activity than opaque cells in the BSA-containing medium (Figure 4A and 4B). The inducible expression mode of *SAP2* could confer advantages to gray cells over white and opaque cells (Figure 4D and 4E). Previous studies have demonstrated that *SAP2* mRNA is highly upregulated in a reconstituted human epithelial (RHE) infection model and critical for epithelial tissue damage [30]–[32]. Constitutive expression of *SAP* genes in opaque cells could cost and waste a lot of energy to the cell, and the damaging effects of Saps could evoke strong host immune response [4],[26]. The expression of *SAP* genes would not favor the commensal life style of *C. albicans*. The low Sap activity of white cells limits certain infection abilities, especially the ability to cause cutaneous infections. Inducible expression of Sap activity could also play a balancing role in the transition between the commensal and pathogenic life styles in *C. albicans*. The major components of the skin surface are proteins, such as collagen, elastin, and keratin. Like BSA, these proteins could also induce Sap activity in gray cells. Consistent with this idea, the skin damage and nutrient acquisition abilities of white, gray, and opaque cells correspond to their respective Sap activities exhibited in BSA-containing media (Figures 4, 5D, and S8). Gray cells and opaque cells are less virulent in the systemic infection model possibly due to their weaker abilities to filament compared to white cells (unpublished data). Gray cells and opaque cells propagated faster than white cells in an ex vivo tongue infection model and led to more serious damages in an in vivo skin infection model (Figure S8). These results are consistent with previous studies in terms of the correlation between Sap activity and cutaneous infections [26].

Moreover, the three cell types differ in fungal burdens in different organs (Figure 5C), suggesting that they may play distinct and specific roles in systemic infections. The distinct global transcriptional profiles and differential Sap activities of the three different cell types may contribute to the adaptability of *C. albicans* to inhabit a diverse number of host niches and to propagate in different tissues.

### Roles of the *MTL* Locus, Wor1, and Efg1 in the Regulation of White-Gray-Opaque Transitions

The *MTL* locus is involved in the regulation of white-opaque switching and sexual mating in *C. albicans* [7]. Here we found that the white-gray-opaque tristable switching system is independent of the regulation of the *MTL* locus. Although only a subset of *C. albicans* clinical strains undergo the tristable switch under the culture conditions tested, we suggest that this phenotypic switching system may be a general feature of natural *C. albicans* strains, and most, if not all, strains could do this under certain conditions (e.g., in certain niches of the host).

Wor1 is the master regulator of white-opaque switching and is essential for opaque cell formation (Figures 6, S9, and S10) [14]–[16]. Efg1 plays a negative role in white-to-opaque switching and is essential for white cell formation (Figures 6 and S10). Wor1 and Efg1, together with transcription factors Czf1, Wor2, and Wor3, form interlocking transcriptional feedback loops controlling white-opaque switching [18]. Although neither Wor1 nor Efg1 is required for the formation of the gray phenotype, deletion of both *WOR1* and *EFG1* locks cells in the gray phenotype (Figures 6, S9, and S10). These results suggest that different transcriptional circuitries may be involved in the regulation of white-gray and gray-opaque transitions. Together with other unidentified regulators, Wor1 and Efg1 may coordinately govern the formation of gray cells. On phloxine B-containing media, the cellular and colony morphologies of the *wor1/wor1 efg1/efg1* double mutant we generated are similar to those of the *wor1/wor1 efg1/efg1* double mutant in the SC5314 background constructed by Hnisz and colleagues [33], suggesting that derivatives of SC5314 also have the potential to switch to the gray phenotype. Both CO_2_ and GlcNAc induce white-to-opaque switching predominantly via activating the Wor1 regulator [21]; however, neither of these stimuli had an obvious effect on the induction of white-to-gray switching (Figure 2). This is reasonable because the expression level of *WOR1* is very low in gray cells and Wor1 is not required for the formation of gray cells (Table S1).

### The Gray Cell Type Differs from the Reported “GUT” Cell Type

Gray cells also differ from the recently reported GUT cell type (gastrointestinally induced transition) in several aspects [4]. First, GUT cells resemble opaque cells in shape and cell size but lack cell wall surface pimples [4]. Gray cells are much smaller than both opaque and GUT cells. Second, GUT cells have been reported to exist only in the animal gut and have not been found to be stable in vitro culture conditions, while gray cells are very stable in a variety of laboratory culture conditions. Third, several virulence genes including *SAPs*, are downregulated in GUT cells, while gray cells have a higher Sap activity than white and opaque cells in the presence of BSA. The switching characteristic, together with the unique aspects of gray cells discussed above, indicates that the gray phenotype is a novel morphological phenotype.

### Is the Gray Cell Type an Intermediate of the White and Opaque Phenotypes?

A number of reasons suggest that the gray cell type is not an intermediate of the white and opaque phenotypes. First, white cells can directly switch to the opaque phenotype under some culture conditions at high frequencies without an intermediate phase (Figure 2), and vice versa. Consistently, the frequencies of white-to-opaque switching is even higher than that of gray-to-opaque switching under certain conditions, suggesting that conversion to the gray phenotype is not necessary to facilitate the formation of opaque cells. Second, the key regulators of white-opaque switching, Wor1 and Efg1, are not required for the maintenance of the gray phenotype. If the gray phenotype were an intermediate of the white and opaque phenotypes, one would expect that the expression levels of white- or opaque-phase specific genes, such as *WOR1*, *EFG1, WH11*, and *OP4* [2], would be at intermediate levels in gray cells. However, this was not the case (Tables S1 and S2). Third, the order of Sap activities from highest to lowest is gray>opaque>white cells. In this sense, the opaque phenotype is an intermediate of the white and gray phenotypes. Fourth, the gray phenotype is not transient, but heritable, and can be maintained for many generations. Fifth, similar to white and opaque cells, gray cells exhibit a unique global gene expression profile.

### Conclusion

Understanding the regulatory mechanisms of phenotypic changes will provide insights into several fundamental questions such as how pathogens adapt to the host and survive and propagate under diverse niches. Here we add the gray morphology and the white-gray-opaque tristable transitions to the list of known fungal morphological switching systems. This study provides an example of multiple stable and heritable switching systems, indicating that the regulation of morphological forms to adapt to environmental changes could be much more elaborate than previously thought. Our study also sheds new insight on the regulatory mechanism of the transition between commensal and pathogenic life styles in *C. albicans*. Exploring the molecular basis of this tristable phenotypic switching system and its role in host adaptation will be important for seeking new strategies to treat infections caused by the major fungal pathogen of humans.

## Materials and Methods

### Culture Conditions, Strains, and Plasmids

The strains used in this study are listed in Table S3. Lee's glucose medium and YPD medium (20 g/l glucose, 20 g/l peptone, 10 g/l yeast extract) were used for routine growth of *C. albicans*. Lee's glucose, Lee's GlcNAc [21],[34], and YPD media were used for phenotypic switching assays. 2% agar was added to the media to make solid nutrient plates. The dye phloxine B (5 µg/ml), which stains opaque colonies dark pink and gray colonies light pink, was added to the solid media. The two plasmids for *WOR1* deletion, pSFS2A-WOR1KOa and pSFS2A-WOR1KOb, were used to delete *WOR1* in BJ1097 as described previously [17]. The two plasmids for *EFG1* deletion, pSFS2A-EFG1KOa and pSFS2A-EFG1KOb, were generated by inserting two fragments containing sequences homologous to the 5′- and 3′-terminus of the *EFG1* gene. The primers used for PCR to generate these fragments are listed in Table S4. To generate the *wor1/wor1 efg1/efg1* double mutant, the two alleles of *EFG1* were deleted in the *wor1/wor1* mutant using the same *EFG1* knockout plasmids.

The same strategy we described previously [17] was used to construct the SAP1p-GFP and SAP2p-GFP reporter strains, BJ1097 was transformed with PCR products of the GFP-caSAT1 fragment (amplified from the template plasmid pNIM1 [35] with GFP reporter primers) (Table S4).

### White-Gray-Opaque Switching Assays

The tristable switching assays were performed similarly to previously described white-opaque bistable switching assays [21]. Briefly, white, gray, or opaque cells from cultures of five days were replated onto agar media and incubated in air or 5% CO_2_ at temperatures indicated in the main text for 4 (at 37°C) or 5 (at 25°C) days of growth. Switching frequency = (number of colonies containing the second or third alternative phenotype/total colony number)×100%. For example, white-to-gray switching frequency = (number of gray colonies plus colonies with gray sections/total colony number)×100%. To verify colony phenotypes, several representative colonies of each type were examined for cellular morphology.

SEM assays were performed as described previously [36]. White, gray, and opaque cells were grown on Lee's GlcNAc medium for 3 days at 25°C and used for SEM assays.

### Mating Assay

We first deleted one allele of the *MTL* locus in the parent strain BJ1097 (*MTL* **a**/α) with the plasmid L23.14 [37], generating BJ1097Na (*MTL* **a**/Δ *Clon^+^*) and BJ1097Nα (*MTL*Δ/α *Clon^+^*). The two strains were then grown in YPmal medium (1% yeast extract, 2% peptone, 2% maltose) for FLP-mediated excision of the *SAT1*/flipper cassette, generating BJ1097a (*MTL* **a**/Δ *Clon^−^*) and BJ1097α (*MTL*Δ/α *Clon^−^*). Experimental strains of the *wor1/wor1, efg1/efg1*, *wor1/wor1 efg1/efg1* double mutants (*MTL*Δ/α, *URA3* ^+^ *Clon^−^*) were generated by using a similar strategy. To generate the *ura3^−^ Clon^+^* tester strain (WTa, opaque in Figure 7B), the plasmid pNIM1, which contains a *caSAT1* gene, was linearized and integrated into GH1012 (*MTL* **a**/**a** *ura3^−^*) [20], generating GH1012N. Quantitative mating assays were performed according to our previous publication [17]. Briefly, the mating experiments were performed on Lee's glucose medium at 25°C. The experimental white, gray, and opaque cell samples were collected from Lee's glucose medium plates. To test the mating efficiencies, 1×10^6^ of GH1012N opaque cells and 1×10^6^ of experimental cells (in white, gray, or opaque phase) were mixed and cultured on Lee's glucose medium plates for 48 hours at 25°C. The mating mixtures were resuspended, diluted, and plated onto three types of selectable plates for growth. Mating efficiencies were calculated as previously described [17].

### Sap Activity Testing Assays

YCB-BSA assay. Sap activity was monitored on YCB medium agar containing 0.2% BSA as the sole nitrogen source as described previously [38]. 5×10^6^ cells of each cell type in 5 µl ddH_2_O were spotted onto the plates. The white halos indicate secreted enzyme activity. The size of the halo ring indicates the activity level. This experiment was repeated six times.

Quantitative Sap activity assays were performed according to Ray and colleagues [39]. Briefly, white, gray, and opaque cells were grown in Lee's glucose or YCB-BSA medium overnight at 25°C. An aliquot of 20 µl of the cell suspension (1×10^6^ cells) was inoculated into 2 ml of fresh Lee's glucose or YCB-BSA medium. After incubation for 48 h at 25°C, the cell number of each sample was determined. The cultures were centrifuged at 13,000 rpm for 1 min. For activity assays, 250 µl of culture supernatant was mixed with 500 µl of 1% BSA in 0.1 M sodium citrate-HCl buffer (pH 3.0). The reaction mixtures were incubated at 37°C for 1 h with gentle agitation. The reaction was stopped by adding 1.25 ml of ice-cold 5% trichloroacetic acid (TCA). Precipitated material was removed by centrifugation and the protein concentration of the supernatant was determined in a modified Bradford assay according to the manufacturer's protocol (Sangon Biotech). The activities were calculated for 10^8^ cells of each cell type. One arbitrary unit was defined as an extinction increase at 595 nm of 0.1/h.

### Virulence Assays

All animal experiments were performed according to the guidelines approved by the Animal Care and Use Committee of the Institute of Microbiology, Chinese Academy of Sciences. The present study was approved by the Committee.

Systemic infection of mice was performed according to the previous studies [17],[36], with slight modifications. Female BALB/c mice aged 4–5 weeks were used for survival and competition experiments. 10 mice were used for injection of each cell type (white, gray, or opaque). 3.75×10^6^ cells of each type were injected into a mouse via tail vein. For competition experiments, 5×10^5^ cells of each type (white, gray, or opaque) of BJ1097 were mixed with 5×10^5^ cells of SC5314N (Nou^R^, locked in white phase) in 250 µl PBS and then injected into a mouse via tail vein. Mice were humanely killed at 24 hours after injection. Different organs were used for fungal burden assays. BJ1097 is sensitive to 100 µg/ml nourseothricin (clonNAT). To generate the nourseothricin resistant strain SC5314N, pNIM1 was linearized and integrated into the laboratory strain SC5314. Organ tissues (liver, kidney, spleen, lung, and brain) were homogenated, diluted in PBS, and plated on Lee's medium for 3 days of growth at 37°C in 5% CO_2_ for colony-forming unit (CFU) calculation. Colony morphologies of SC5314N and BJ1097 were distinguishable. SC5314N formed wrinkled and filamentous colonies, while BJ1097 formed smooth colonies under this culture condition. SC5314N and BJ1097 were also subject to the nourseothricin susceptibility test. Both colony morphology and nourseothricin susceptibility assays were used to distinguish SC5314N and BJ1097 formed colonies. Competitive index = the ratio of CFU number of BJ1097 to CFU number of SC5314N in each organ.

Skin infection assays were performed as described previously [17], with modifications. Newborn BALB/c mice (aged 2–4 days) were used. 4×10^6^ cells of each cell type in 2 µl ddH_2_O were spotted on the skin on the back of a new born mouse. After water evaporated, a small sterile filter paper was covered and fixed on the fungal spot with First Aid tape. After 24 h, the infected areas were excised for SEM assays.

Ex vivo tongue infection assays were performed as described by Kamai and colleagues [40], with modifications. Tongues (of similar size and weight) were excised from humanely killed female BALB/c mice aged 4–5 weeks and added to each well of a 24-well polystyrene plate containing 1×10^7^ cells of white, gray, or opaque cells in 1 ml PBS. 50 µg/ml ampicillin and 50 µg/ml kanamycin were added to each well to inhibit bacterial growth. After 24 hours of incubation at 37°C, cells in the liquid and on the tongue (after homogenization) were separately plated onto YPD agar for CFU assays. The total cell number of each well (including cells in the liquid and attached to the tongue) was calculated. The total cell number of each well indicates fungal growth rate. The experiment was repeated three times. For each time, three tongues were used for each cell type.

### RNA Etraction and RNA-Seq

White, gray, and opaque cells were grown at 25°C in liquid Lee's glucose medium for 24 h and total RNA was extracted using GeneJET RNA Purification kits according to the manufacturer's instructions. RNA-Seq analysis was performed by the company BGI-Shenzhen according to the company's protocol (http://www.genomics.cn/) [41]. Approximately 10 million (M) reads were obtained by sequencing each library. The library products were sequenced using the Illumina HiSeq 2000. Software Illumina OLB_1.9.4 was used for basecalling. The raw reads were filtered by removing the adapter and low quality reads (the percentage of low quality bases with a quality value ≤5 was >50% in a read). Clean reads were mapped to the genome of *C. albicans* SC5314 using SOAP aligner/soap2 software (version 2.21) [42]. The gene expression level is calculated using the RPKM method [43].

### Accession Number

The RNA-seq dataset has been deposited into the NCBI Gene Expression Omnibus (GEO) portal (accession number GSE53671).

## Supporting Information

Figure S1 **Colony and cellular morphologies of white, gray, and opaque cell types on Lee's glucose medium.** G, gray; O, opaque; W, white. Scale bar, 10 µm. Cells (strain BJ1097) were grown at 25°C in air for 5 days. (A) Colony and cellular morphologies of white, gray, and opaque cell types. (B) An image of mixed colonies of white, gray, and opaque cell types. (C) Fluorescence activated cell sorting (FACS) analysis of the DNA content of white, gray, and opaque cells. The strain SC5314 served as a diploid control. Cells were grown in liquid Lee's glucose medium at 25°C for 18 hours. The y-axis represents cell count, and the x-axis indicates the fluorescence intensity of the nuclear DNA.
(TIF)

Figure S2 **Colony and cellular morphologies of white, gray, and opaque cell types on Lee's GlcNAc medium.** G, gray; O, opaque; W, white. Scale bar, 10 µm. Cells (strain BJ1097) were grown at 25°C in air for 5 days. (A) Colony and cellular morphologies of white, gray, and opaque cell types. (B) An image of mixed colonies of white, gray, and opaque cell types.
(TIF)

Figure S3 **Cellular morphology of white, gray, and opaque cell types in liquid Lee's medium.** Scale bar, 10 µm. Cells (strain BJ1097) were grown at 25°C in liquid Lee's glucose medium with shaking for 8 to 48 hours and imaged.
(TIF)

Figure S4 **Scanning electron microscope images of white, gray, and opaque cells of** ***C. albicans*** **.** Scale bar, 3 µm. Cells (strain BJ1097) grown at 25°C in Lee's GlcNAc medium for 3 days were used for SEM assays. A pimpled- (left) and a smooth-surfaced (right) opaque cell are shown.
(TIF)

Figure S5 **Cellular morphology of white, gray, and opaque cells of** ***C. albicans*** **at 37°C.** Scale bar, 10 µm. Cells (strain BJ1097) were plated onto solid media indicated and grown at 37°C for 4 days. Cellular images of representative colonies are shown.
(TIF)

Figure S6 **White-gray-opaque tristable transitions in 5% CO_2_.** G, gray; G-sec, gray sectors; O, opaque; W, white. Colonies (strain BJ1097) were grown on YPD medium plates in 5% CO_2_ at 25°C for 5 days. Cellular images of representative colonies are shown. Scale bar, 10 mm. Switching frequencies are shown in Figure 2C.
(TIF)

Figure S7 **White-gray-opaque tristable transitions in six independent clinical isolates of** ***C. albicans*** **.** JX1345, JX1346, and JX1352 were isolated from China; GH1501 and 19F were isolated from the United States; and GH1526 was isolated from Spain. Cells were plated onto Lee's medium agar and grown at 25°C for 5 days. Cellular morphologies of the white, gray, and opaque phenotypes are shown. Scale bar, 10 µm.
(TIF)

Figure S8 **SEM images of infected skin samples of new born mice.** Damaged regions are indicated with white arrows. 4×10^6^ cells of each type in 2 µl ddH_2_O were spotted on the skin on the back of a new born mouse. After water evaporated, a small sterile filter paper was affixed on the fungal spot with First Aid tape. After 24 h, the infected areas were excised for SEM assays. Uninfected, a sample of uninfected skin tissue. Scale bar, 5 µm. Rectangular marks in some panels occurred during routine focusing procedures due to a longer exposure time to the electron probe.
(TIF)

Figure S9 **Colony (A) and cellular (B) morphologies of the WT,** ***wor1/wor1*** **,** ***efg1/efg1*** **, and** ***wor1/wor1 efg1/efg1*** **double mutants.** Cells were grown on Lee's GlcNAc medium plates at 25°C in air for 4 days. Cellular morphology of a representative colony of each cell type is shown. G, gray; G-sec, gray sectors; O, opaque; W, white. Scale bar, 10 µm.
(TIF)

Figure S10 **Switching frequencies in the** ***wor1/wor1*** **,** ***efg1/efg1*** **, and** ***wor1/wor1 efg1/efg1*** **double mutants.** G, gray; O, opaque; W, white. Cells were grown under the conditions indicated in the figure for five days. Colonies were counted for switching frequency (%) calculations. (A) Switching frequencies of the *wor1/wor1* mutant in air. (B) Switching frequencies of the *wor1/wor1* mutant in 5% CO_2_. (C) Switching frequencies of the *efg1/efg1* mutant in air. (D) Switching frequencies of the *efg1/efg1* mutant in 5% CO_2_. (E) Switching frequencies of the *wor1/wor1 efg1/efg1* double mutant in air. (F) Switching frequencies of the *wor1/wor1 efg1/efg1* double mutant in 5% CO_2_.
(TIF)

Table S1 **Dataset of RNA-Seq analysis of white, gray, and opaque cells.** White-, gray-, opaque-enriched genes, white-gray, white-opaque, and gray-opaque differentially expressed genes are listed in separate sheets in the dataset. Expression profiles of all genes in the three cell types are also shown.
(XLSX)

Table S2 **Functional categories of differentially expressed genes in white, gray, and opaque cells.**
(XLS)

Table S3 **Strains used in this study.**
(DOC)

Table S4 **Primers used in this study.**
(DOC)

## Abbreviations

BSA: bovine serum albumin
CFU: colony-forming unit
GFP: green fluorescent protein
GlcNAc: N-acetylglucosamine
MTL: mating type-like locus
RPKM: reads per kilobase per million
Sap: secreted aspartyl proteinase
SEM: scanning electron microscopy
WT: wild type
YCB: yeast carbon base
YPD: yeast extract-peptone-dextrose
