# Supplementary figures and images for: Discovery of a “White-Gray-Opaque” Tristable Phenotypic Switching System in Candida albicans: Roles of Non-genetic Diversity in Host Adaptation
Source: PLoS Biol. 2014 Apr 1;12(4):e1001830. doi: 10.1371/journal.pbio.1001830 (PMC3972085; doi:10.1371/journal.pbio.1001830)

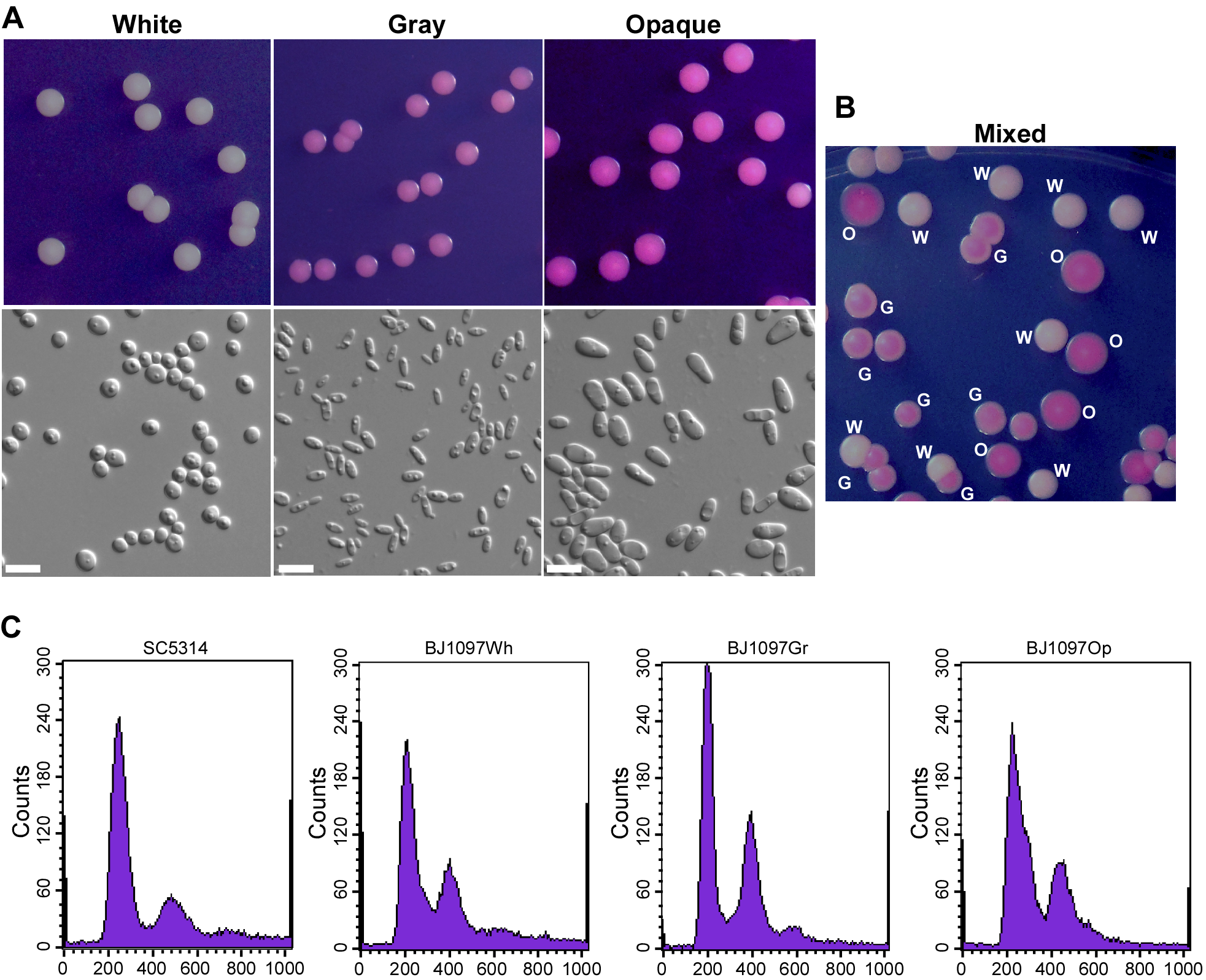

Supplement: Figure S1 — Colony and cellular morphologies of white, gray, and opaque cell types on Lee's glucose medium. G, gray; O, opaque; W, white. Scale bar, 10 µm. Cells (strain BJ1097) were grown at 25°C in air for 5 days. (A) Colony and cellular morphologies of white, gray, and opaque cell types. (B) An image of mixed colonies of white, gray, and opaque cell types. (C) Fluorescence activated cell sorting (FACS) analysis of the DNA content of white, gray, and opaque cells. The strain SC5314 served as a diploid control. Cells were grown in liquid Lee's glucose medium at 25°C for 18 hours. The y-axis represents cell count, and the x-axis indicates the fluorescence intensity of the nuclear DNA. (TIF) [file pbio.1001830.s001.tif]

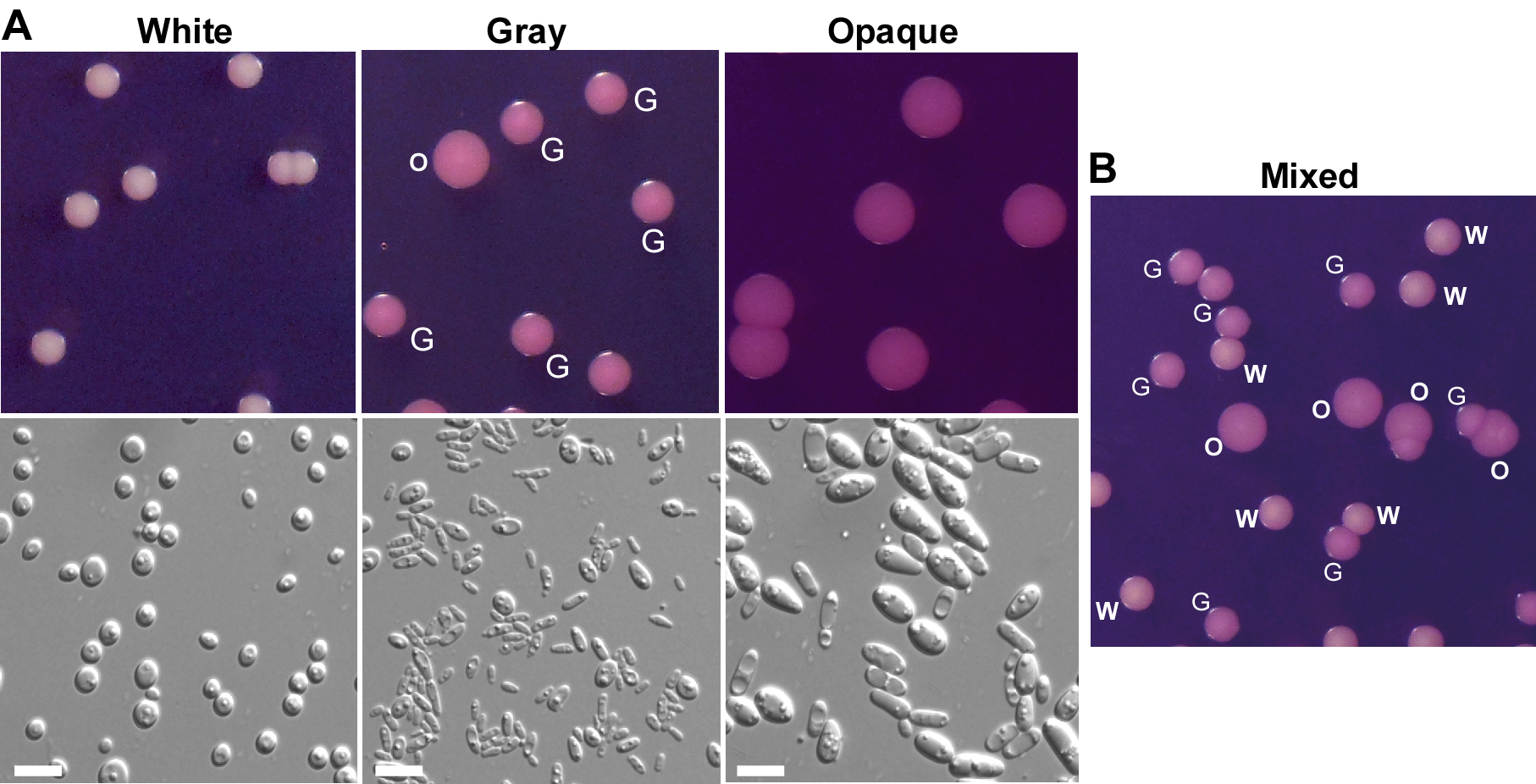

Supplement: Figure S2 — Colony and cellular morphologies of white, gray, and opaque cell types on Lee's GlcNAc medium. G, gray; O, opaque; W, white. Scale bar, 10 µm. Cells (strain BJ1097) were grown at 25°C in air for 5 days. (A) Colony and cellular morphologies of white, gray, and opaque cell types. (B) An image of mixed colonies of white, gray, and opaque cell types. (TIF) [file pbio.1001830.s002.tif]

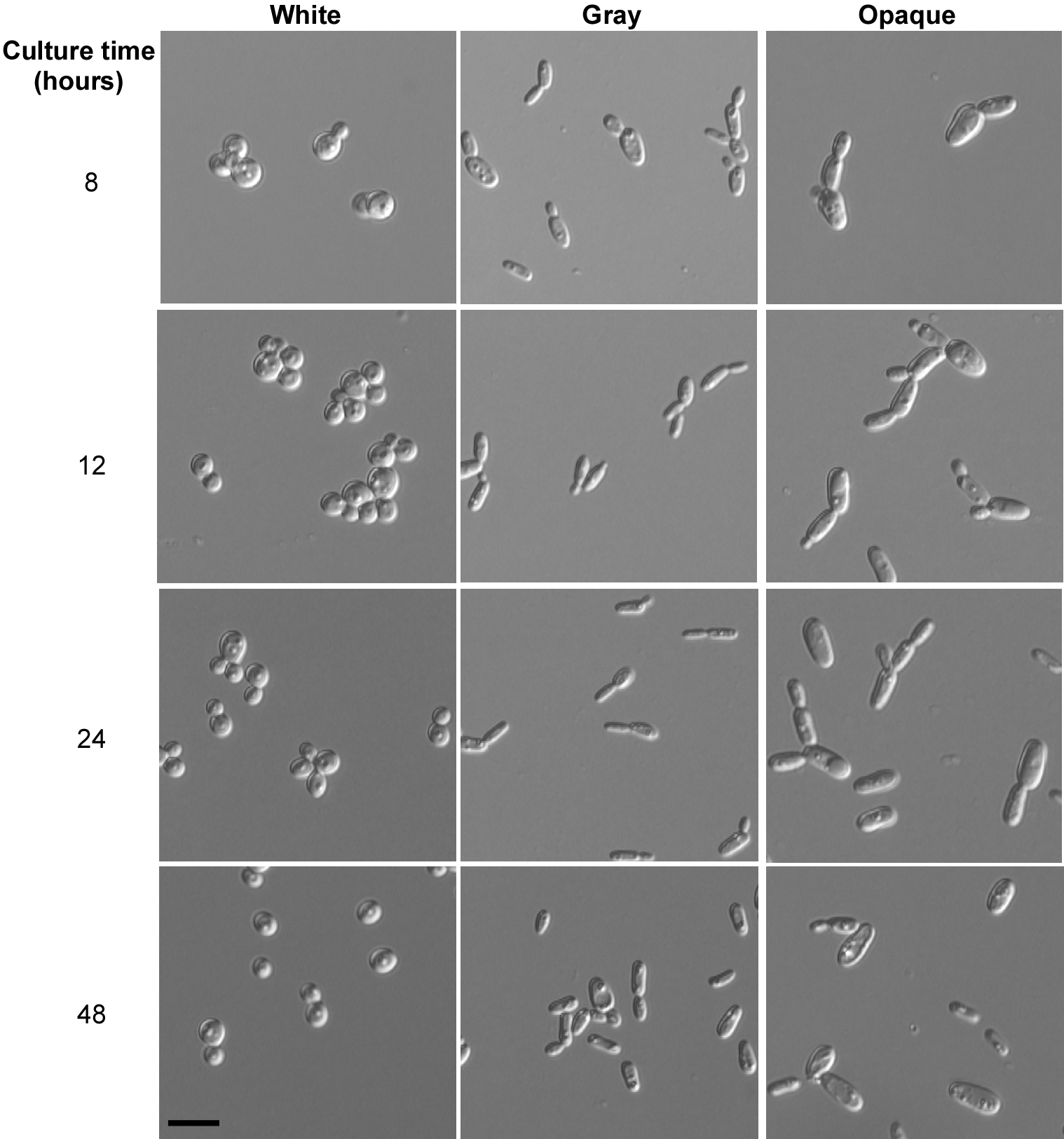

Supplement: Figure S3 — Cellular morphology of white, gray, and opaque cell types in liquid Lee's medium. Scale bar, 10 µm. Cells (strain BJ1097) were grown at 25°C in liquid Lee's glucose medium with shaking for 8 to 48 hours and imaged. (TIF) [file pbio.1001830.s003.tif]

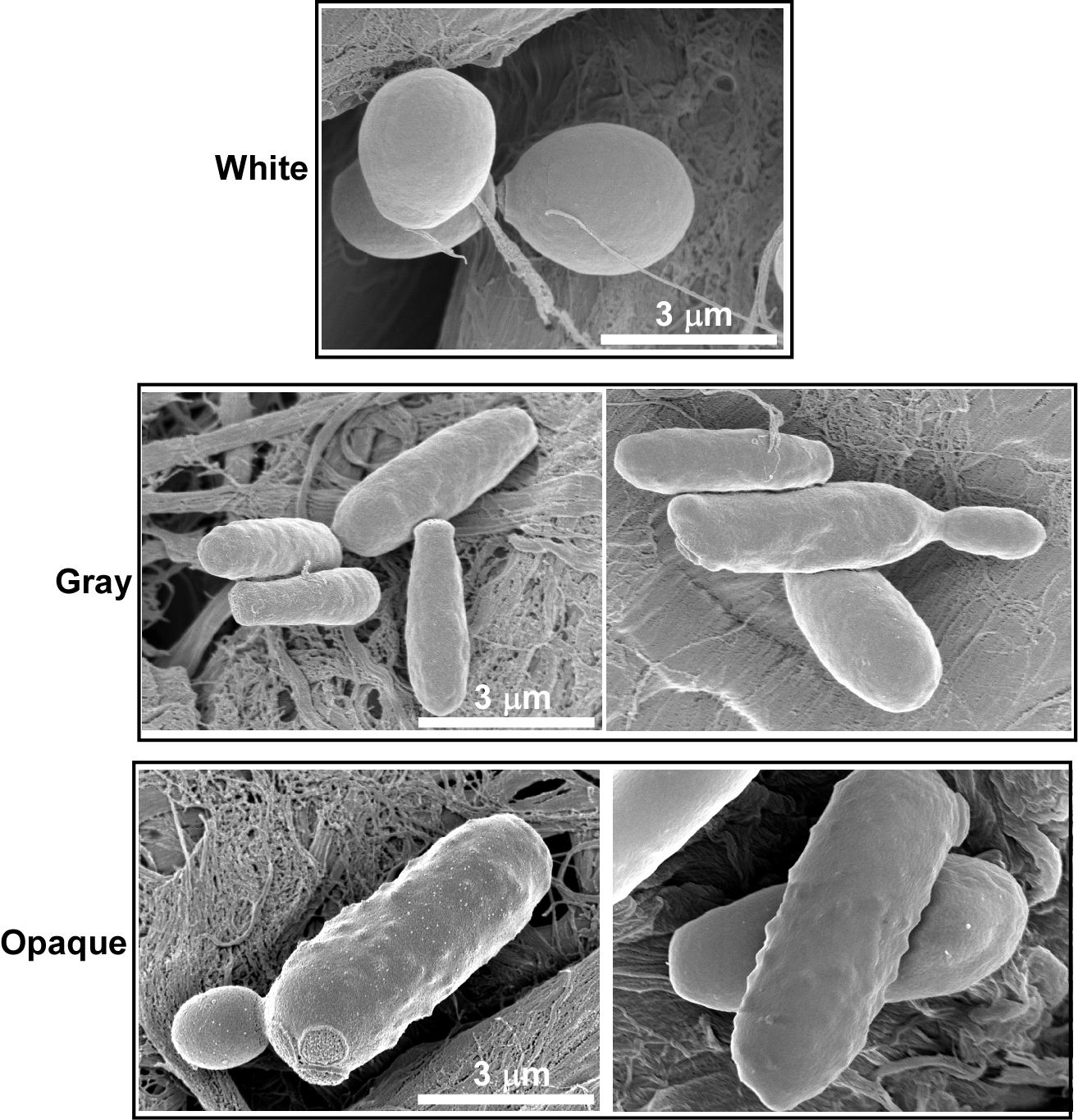

Supplement: Figure S4 — Scanning electron microscope images of white, gray, and opaque cells of C. albicans . Scale bar, 3 µm. Cells (strain BJ1097) grown at 25°C in Lee's GlcNAc medium for 3 days were used for SEM assays. A pimpled- (left) and a smooth-surfaced (right) opaque cell are shown. (TIF) [file pbio.1001830.s004.tif]

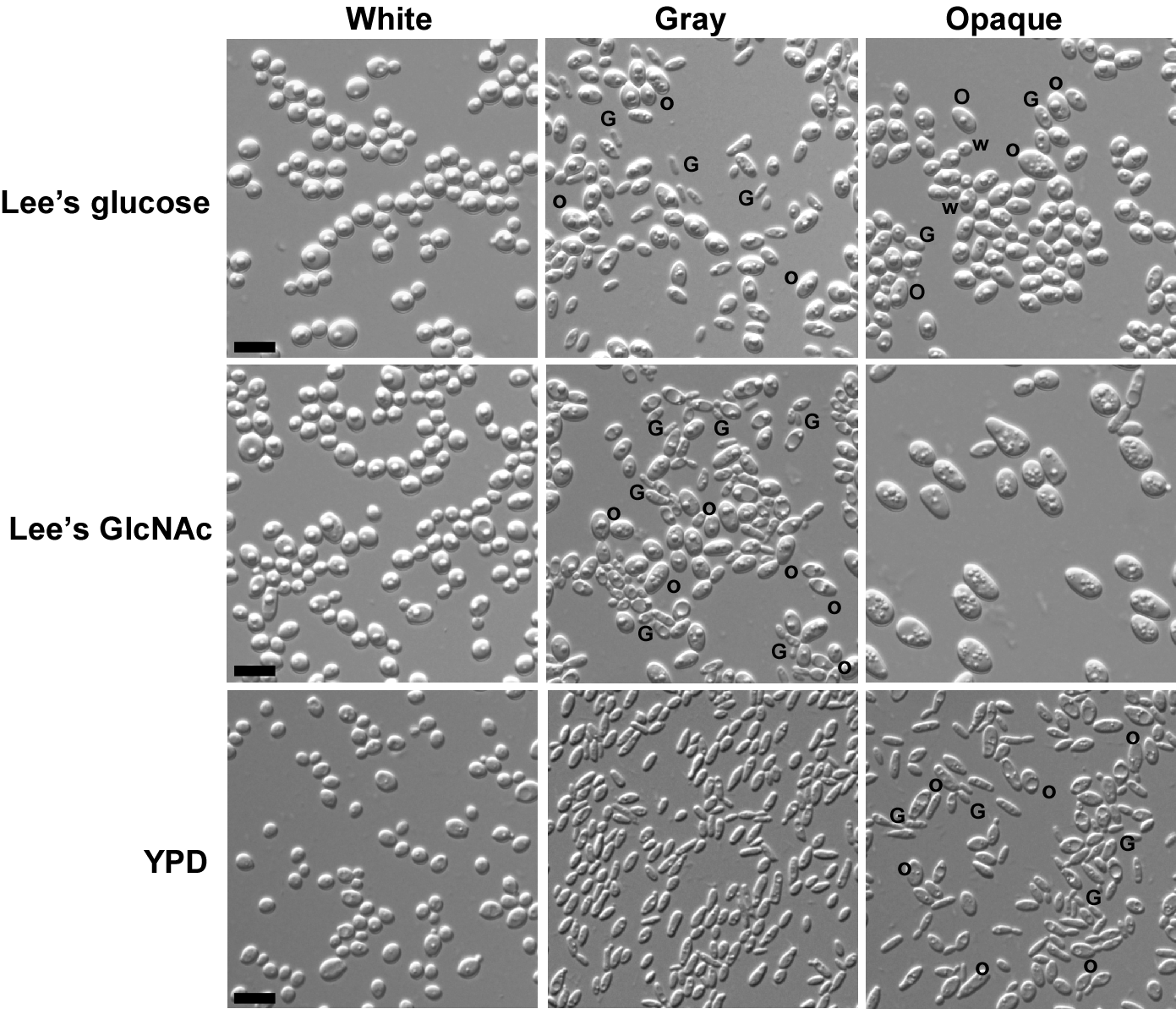

Supplement: Figure S5 — Cellular morphology of white, gray, and opaque cells of C. albicans at 37°C. Scale bar, 10 µm. Cells (strain BJ1097) were plated onto solid media indicated and grown at 37°C for 4 days. Cellular images of representative colonies are shown. (TIF) [file pbio.1001830.s005.tif]

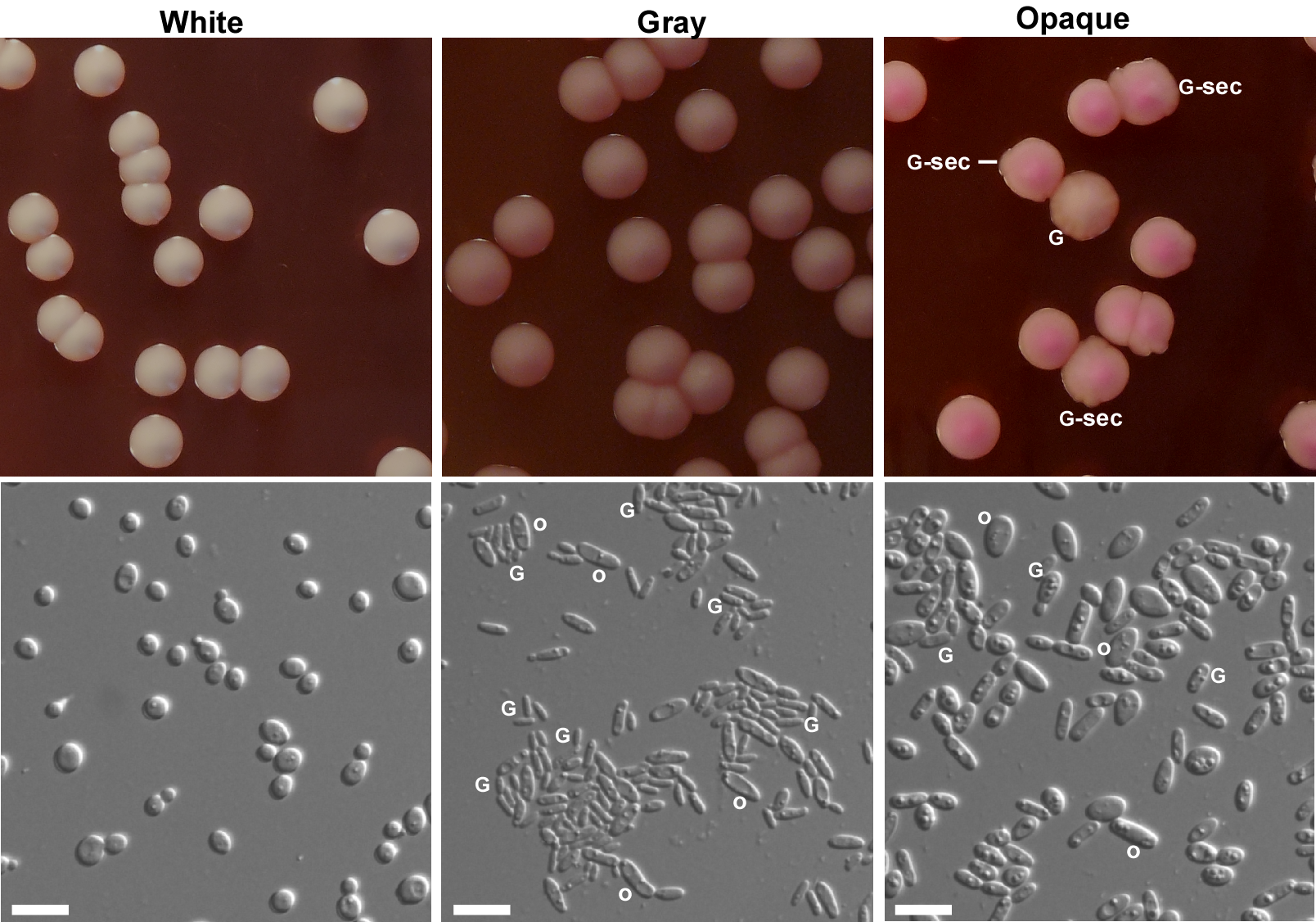

Supplement: Figure S6 — White-gray-opaque tristable transitions in 5% CO2. G, gray; G-sec, gray sectors; O, opaque; W, white. Colonies (strain BJ1097) were grown on YPD medium plates in 5% CO2 at 25°C for 5 days. Cellular images of representative colonies are shown. Scale bar, 10 mm. Switching frequencies are shown in Figure 2C. (TIF) [file pbio.1001830.s006.tif]

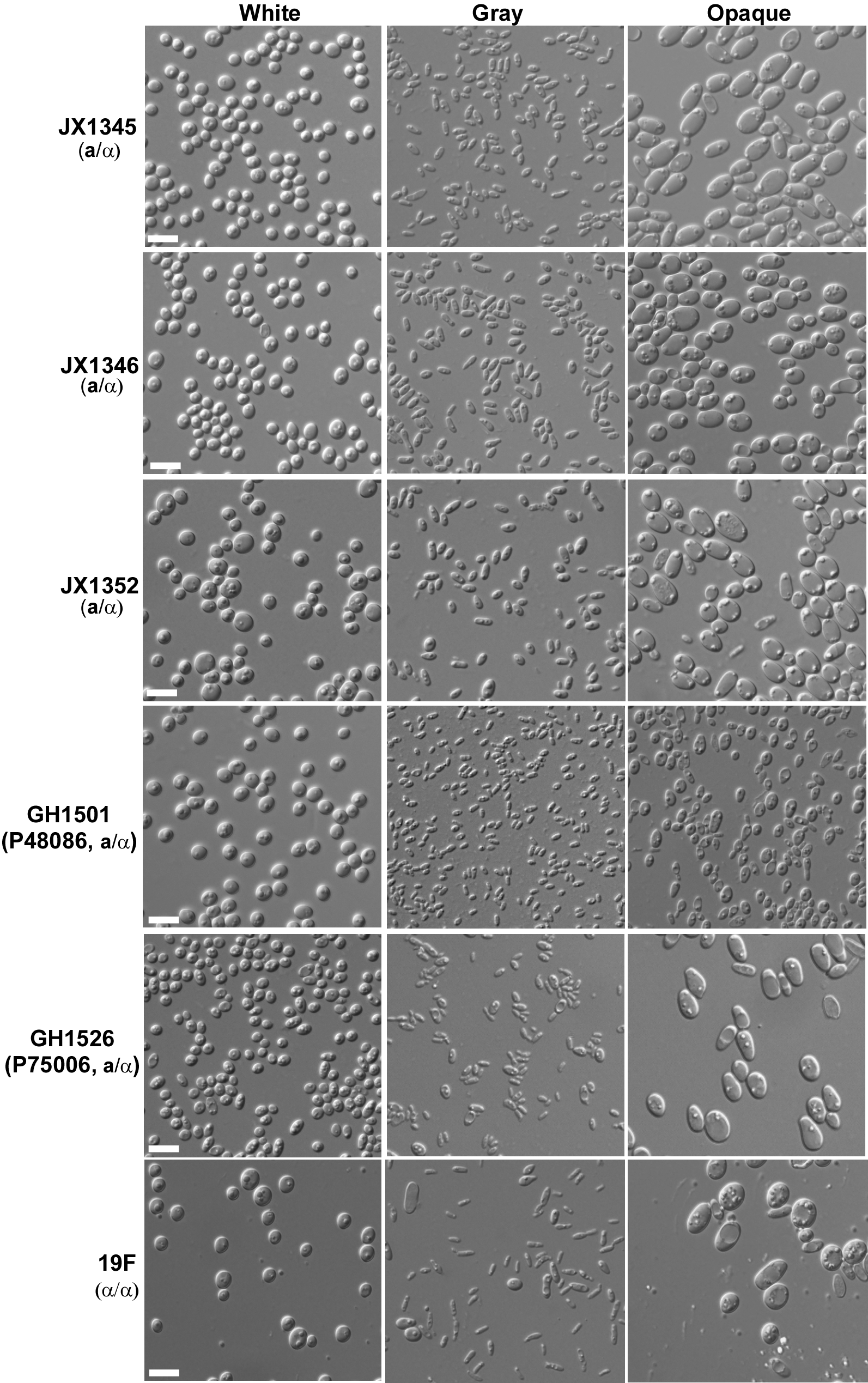

Supplement: Figure S7 — White-gray-opaque tristable transitions in six independent clinical isolates of C. albicans . JX1345, JX1346, and JX1352 were isolated from China; GH1501 and 19F were isolated from the United States; and GH1526 was isolated from Spain. Cells were plated onto Lee's medium agar and grown at 25°C for 5 days. Cellular morphologies of the white, gray, and opaque phenotypes are shown. Scale bar, 10 µm. (TIF) [file pbio.1001830.s007.tif]

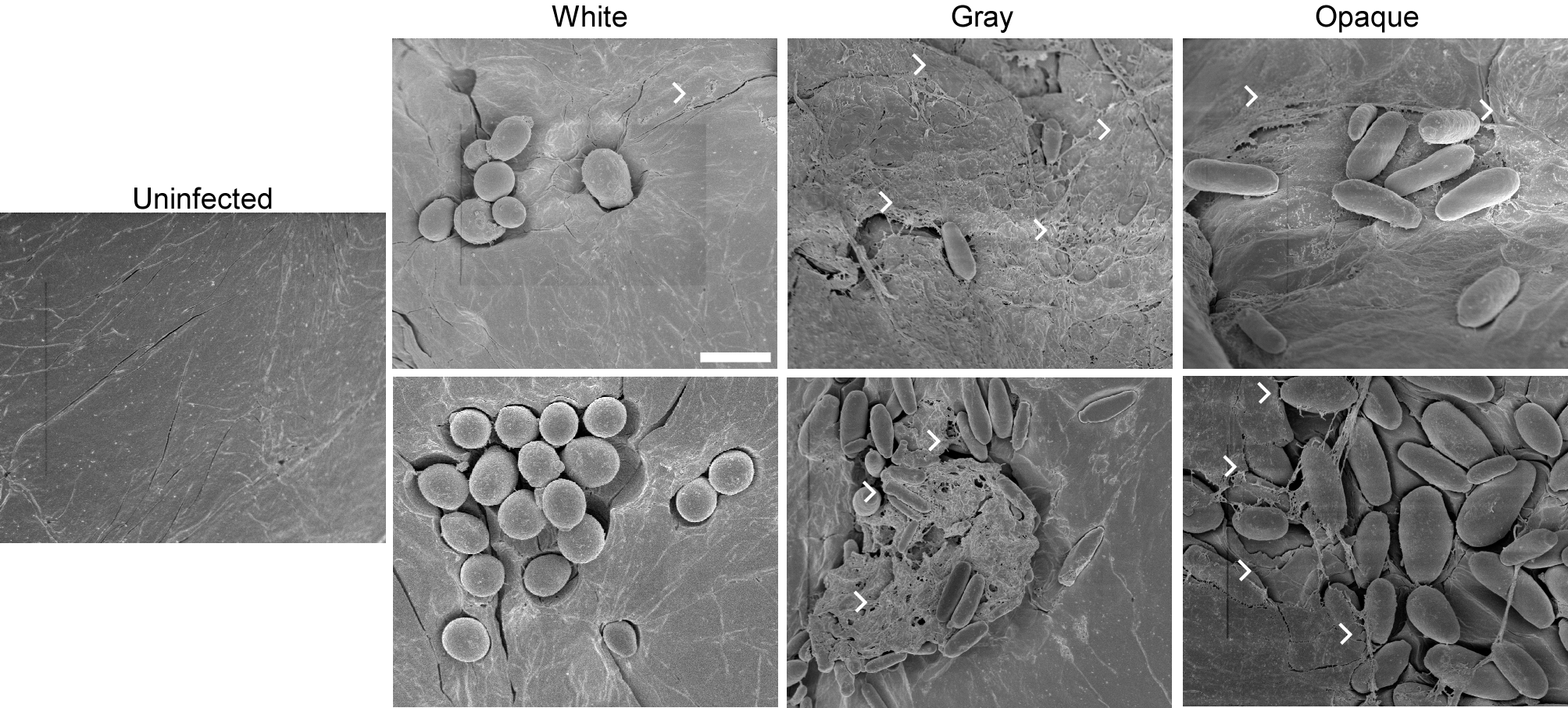

Supplement: Figure S8 — SEM images of infected skin samples of new born mice. Damaged regions are indicated with white arrows. 4×10^6^ cells of each type in 2 µl ddH2O were spotted on the skin on the back of a new born mouse. After water evaporated, a small sterile filter paper was affixed on the fungal spot with First Aid tape. After 24 h, the infected areas were excised for SEM assays. Uninfected, a sample of uninfected skin tissue. Scale bar, 5 µm. Rectangular marks in some panels occurred during routine focusing procedures due to a longer exposure time to the electron probe. (TIF) [file pbio.1001830.s008.tif]

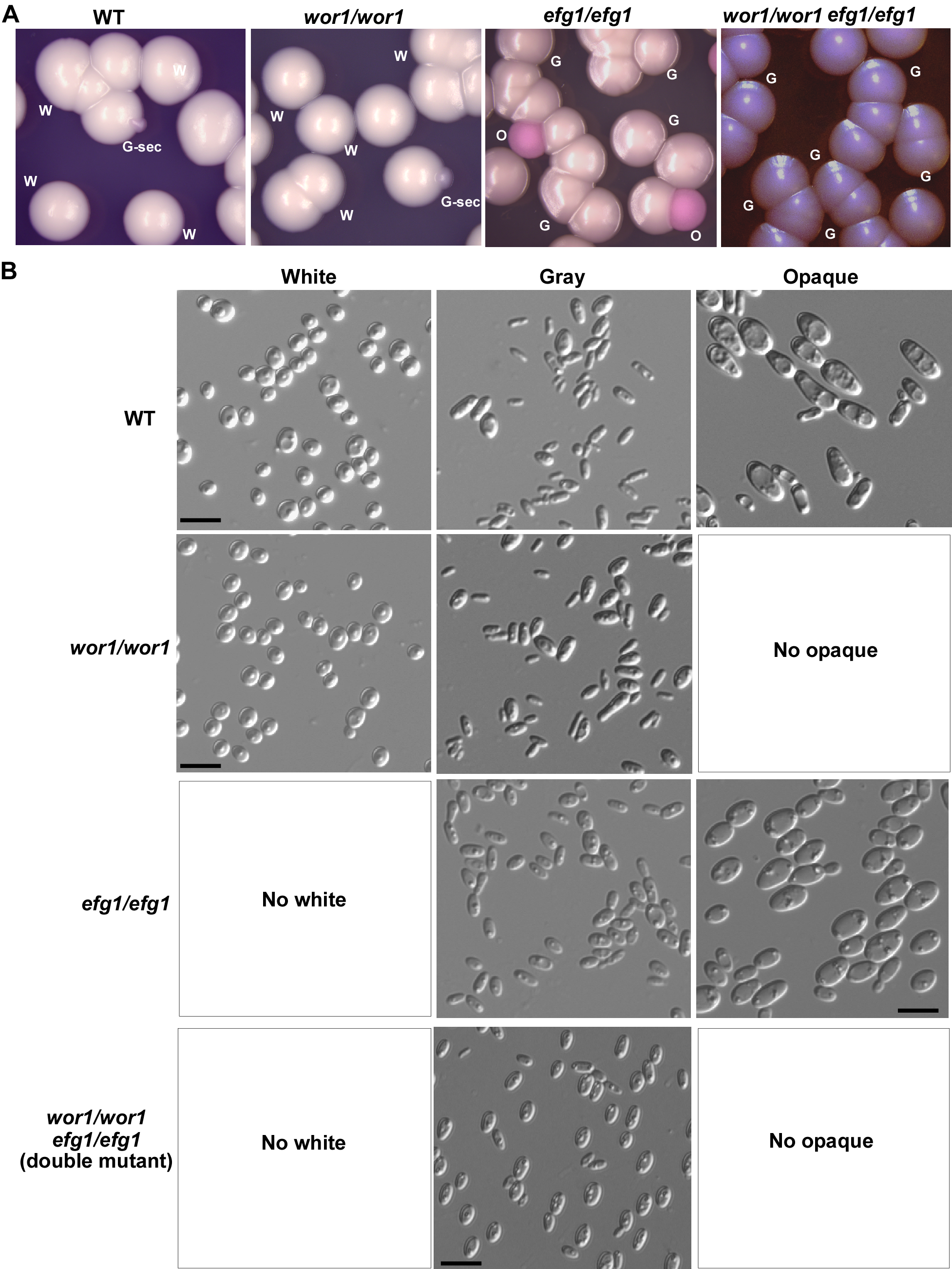

Supplement: Figure S9 — Colony (A) and cellular (B) morphologies of the WT, wor1/wor1 , efg1/efg1 , and wor1/wor1 efg1/efg1 double mutants. Cells were grown on Lee's GlcNAc medium plates at 25°C in air for 4 days. Cellular morphology of a representative colony of each cell type is shown. G, gray; G-sec, gray sectors; O, opaque; W, white. Scale bar, 10 µm. (TIF) [file pbio.1001830.s009.tif]

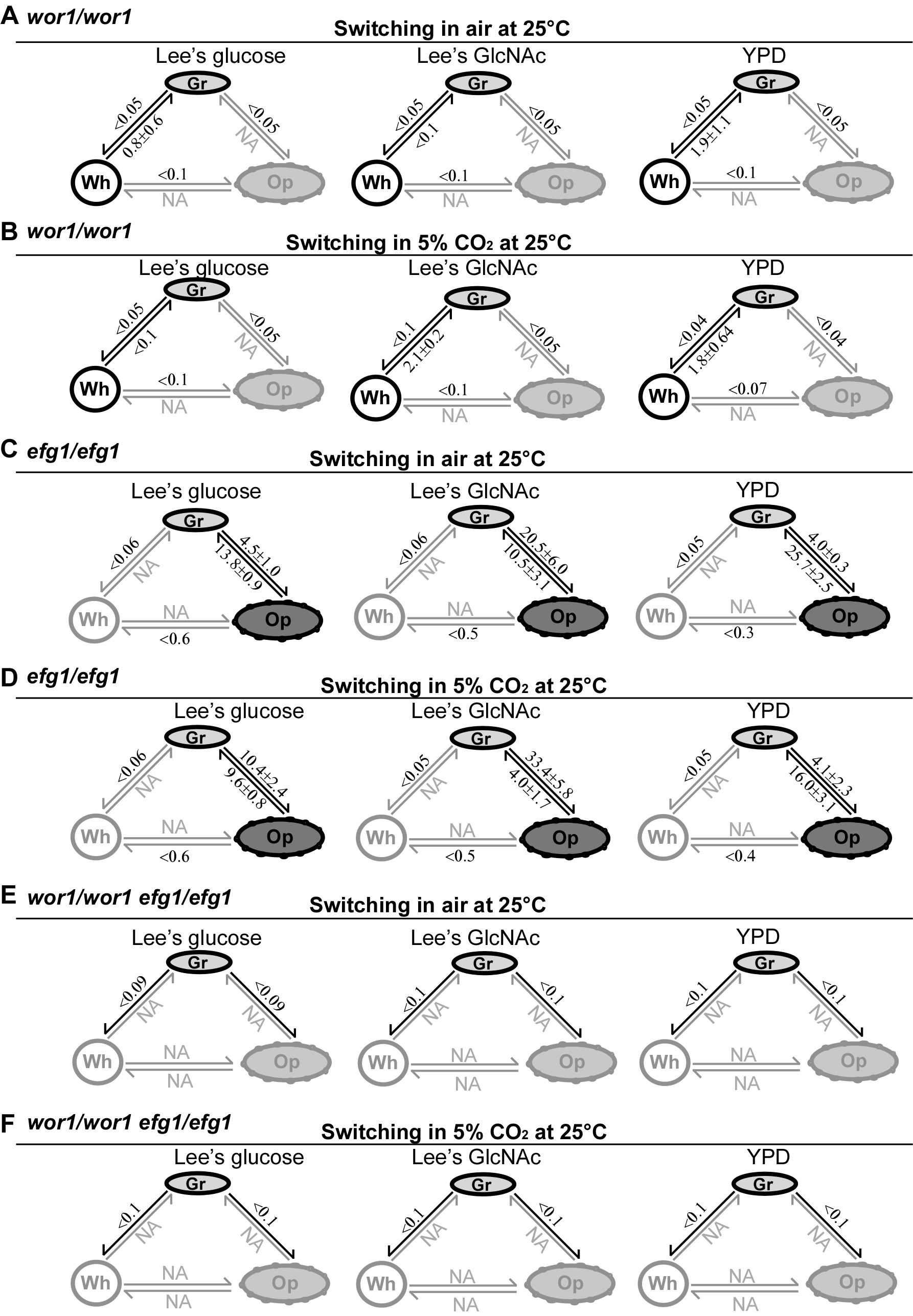

Supplement: Figure S10 — Switching frequencies in the wor1/wor1 , efg1/efg1 , and wor1/wor1 efg1/efg1 double mutants. G, gray; O, opaque; W, white. Cells were grown under the conditions indicated in the figure for five days. Colonies were counted for switching frequency (%) calculations. (A) Switching frequencies of the wor1/wor1 mutant in air. (B) Switching frequencies of the wor1/wor1 mutant in 5% CO2. (C) Switching frequencies of the efg1/efg1 mutant in air. (D) Switching frequencies of the efg1/efg1 mutant in 5% CO2. (E) Switching frequencies of the wor1/wor1 efg1/efg1 double mutant in air. (F) Switching frequencies of the wor1/wor1 efg1/efg1 double mutant in 5% CO2. (TIF) [file pbio.1001830.s010.tif]
