# Supplementary material for: Discovery of a “White-Gray-Opaque” Tristable Phenotypic Switching System in Candida albicans: Roles of Non-genetic Diversity in Host Adaptation
Source: PLoS Biol. 2014 Apr 1;12(4):e1001830. doi: 10.1371/journal.pbio.1001830 (PMC3972085; doi:10.1371/journal.pbio.1001830)
Supplement: Table S3 — Strains used in this study. (DOC) [file pbio.1001830.s013.doc]

**Table S3. Strains used in this study**

| **Strain** | **Parent strain** | **Genotype** | **Reference** |
| --- | --- | --- | --- |
| BJ1097 |  | Wild type, a clinical isolate of *C. albicans* from China, *MTL***a**/α | This study |
| JX1345 |  | Wild type, a clinical isolate of *C. albicans* from China, *MTL***a**/α | 1 |
| JX1346 |  | Wild type, a clinical isolate of *C. albicans* from China, *MTL***a**/α | 1 |
| JX1352 |  | Wild type, a clinical isolate of *C. albicans* from China, *MTL***a**/α | 1 |
| GH1501 (P48086) |  | Wild type, a clinical isolate of *C. albicans* from the United States, *MTL***a**/α | 1,2 |
| GH1526 (P75006) |  | Wild type, a clinical isolate of *C. albicans* Spain, *MTL***a**/α | 1,3 |
| 19F |  | Wild type, a clinical isolate of *C. albicans* from the United States, *MTL*α/α | 3 |
| BJ1097**a** |  | As BJ1097, but *MTL***a***::FRT-SAT1-FRT/*α | This study |
| BJ1097α |  | As BJ1097, but *MTL***a***/*α*::FRT-SAT1-FRT* | This study |
| *wor1/wor1* | BJ1097 | As BJ1097, but *wor1:FRT/wor1::FRT-SAT1-FRT* | This study |
| *efg1/efg1* | BJ1097 | As BJ1097, but *efg1:FRT/efg1::FRT-SAT1-FRT* | This study |
| *wor1/wor1 efg1/efg1* (1) | *wor1/wor1* | As *wor1/wor1*, but *efg1:FRT/efg1::FRT-SAT1-FRT* | This study |
| *wor1/wor1 efg1/efg1* (2) | *wor1/wor1 efg1/efg1* (1) | As *wor1/wor1*, but *efg1:FRT/efg1::FRT* | This study |
| SAP1p-GFP | BJ1097 | As BJ1097, but *SAP1/sap1::SAP1p-GFP* | This study |
| SAP2p-GFP | BJ1097 | As BJ1097, but *SAP2/sap2::SAP2p-GFP* | This study |

**References:**

1. Xie J, Tao L, Nobile CJ, Tong Y, Guan G, Sun Y, *et al*. (2013) White-opaque switching in natural MTLa/α isolates of *Candida albicans*: evolutionary implications for roles in host adaptation, pathogenesis, and sex. PLoS Biol. 2013;11(3):e1001525.

2. Lockhart SR, Pujol C, Daniels KJ, Miller MG, Johnson AD, *et al.* (2002) In *Candida albicans*, white-opaque switchers are homozygous for mating type. Genetics 162: 737-745.

3. Pujol C, Pfaller M, Soll DR (2002) Ca3 fingerprinting of Candida albicans bloodstream isolates from the United States, Canada, South America, and Europe reveals a European clade. J Clin Microbiol 40: 2729-2740.
