# Supplementary material for: Discovery of a “White-Gray-Opaque” Tristable Phenotypic Switching System in Candida albicans: Roles of Non-genetic Diversity in Host Adaptation
Source: PLoS Biol. 2014 Apr 1;12(4):e1001830. doi: 10.1371/journal.pbio.1001830 (PMC3972085; doi:10.1371/journal.pbio.1001830)
Supplement: Table S4 — Primers used in this study. (DOC) [file pbio.1001830.s014.doc]

**Table S4. Primers used in this study**

| **Name** | **Sequence (5’ to 3’)** | **Purpose** |
| --- | --- | --- |
| Wor1CHF | CAACAACAACAACAACAACACC | *WOR1* deletion confirmation  (pair 1) |
| Wor1CHR | TGCCATTACCACCACTAACAC |
| Wor1CHF5 | CACACTATACAAGAAGAGAAACC | *WOR1* deletion confirmation  (pair 2) |
| pSFR | CAATGAAATCCAGACAGTCGAG |
| pSFF | CGATTAGAGACACAAACGAAC | *WOR1* deletion confirmation  (pair 3) |
| Wor1CHR3 | GAATTAGGGTTATGGTATGATG |
| LT362 | atggtttcagctgctatactgc | *WOR1* deletion confirmation  (pair 4) |
| LT363 | atgatgattctgtttgaggtgg |
| SF-EFG5F1 | TTATATGGGCCCCATTTAGCTGCTATTTCAACC | For EFG1 knockout plasmid pSFS2A-EFG1KOa |
| SF-EFG5R1 | AATCATCTCGAGAGTTAAGTGGGTTGGCTGGATG |
| SF-EFG3F1 | TAATATCCGCGGGAGATGATAGTTTGTGGCGTG |
| SF-EFG3R1 | ATATACGAGCTCATCACCTGTAACTCGTGTCG |
| SF-EFG5F2 | TTATATGGGCCCGAAGAGACAAGCAAACAAACG | For EFG1 knockout plasmid pSFS2A-EFG1KOb |
| SF-EFG5R2 | AATCATCTCGAGTGGGTTATATTCTTGGTAGTC |
| SF-EFG3F2 | TAATATCCGCGGGTTCAGTTCACCCTTCACC |
| SF-EFG3R2 | ATATACGAGCTCAAGACAATGACTTACACAC |
| EFG1CHF5 | TATCCCAACTTTAATTCCTTCC | *EFG1* deletion confirmation  (pair 1) |
| pSFR | CAATGAAATCCAGACAGTCGAG |
| pSFF | CGATTAGAGACACAAACGAAC | *EFG1* deletion confirmation  (pair 2) |
| EFG1CHR3 | TGATGATACGGTTAGAAGTC |
| EFG1CHF | CAACAACAAGGACAACCAGG | *EFG1* deletion confirmation  (pair 3) |
| EFG1CHR | AACCGACACATTATTGGCATC |
| SAP1pGFP-F | tgaaataaatcatatttaatccaacaatcaatcaattcactcttccatttctaacaaacaAATATAAATAGTCGACAAAG | SAP1p-GFP reporter |
| SAP1pGFP-R | AAGAAAGACCTAAAAGGATGGCATTTTAATCGACACTTATAACCTAAAAAGTAGGTATCTgaccacctttgattgtaaatag |
| SAP2pGFP-F | ataatatcaatcaattaatcaatcaaataacaacaacccactaaacatcaccatttatcaAATATAAATAGTCGACAAAG | SAP2p-GFP reporter |
| SAP2pGFP-R | AGTAAAAACTAATCAAGCAACTAATATTTTAATATTTTAACTTTATTCCACCCCTTCATCgaccacctttgattgtaaatag |
| SAP1pGFP-DF | TCGGTCACATATAACTACAG | GFP reporter checking |
| SAP2pGFP-DF | AACCTGGAAATCGGGGAAAC |
| GFP-DR | AGCATTGAAGACCATACGCG |
